# *C*-Locked Analogs of the Antimicrobial Peptide BP214

**DOI:** 10.3390/antibiotics11081080

**Published:** 2022-08-09

**Authors:** Ida Kristine Lysgaard Andersen, Thomas T. Thomsen, Jasmina Rashid, Thomas Rønnemoes Bobak, Alberto Oddo, Henrik Franzyk, Anders Løbner-Olesen, Paul R. Hansen

**Affiliations:** 1 Department of Drug Design and Pharmacology, Faculty of Health and Medical Sciences, University of Copenhagen, Universitetsparken 2, 2100 Copenhagen, Denmark; 2 Department of Clinical Microbiology, Rigshospitalet, Henrik Harpestrengs Vej 4A, 2100 Copenhagen, Denmark; 3 Department of Biology, Section for Functional Genomics, University of Copenhagen, Ole Maaløes Vej 5, 2200 Copenhagen, Denmark

**Keywords:** antimicrobial peptides, bacterial killing, BP214, cyclic lipopeptides

## Abstract

BP214 is an all-D antimicrobial peptide amide, kklfkkilryl, which shows an excellent activity against colistin-resistant *Acinetobacter baumannii* and a low hemolytic activity. The aim of the present work was to investigate how *C*-terminus-to-side chain macrocyclization and fatty acid modification affect the antimicrobial and hemolytic activity of this peptide. In total, 18 analogs of BP214 were synthesized using a combination of Fmoc-based solid-phase peptide synthesis and the submonomer approach. Cyclization was achieved by reacting the ε-amino group of a *C*-terminal lysine residue with a bromoacetylgroup attached to the *N^α^* amino group of the N-terminal amino acid, generating a secondary amine at which the exocyclic lipopeptide tail was assembled. Three different ring sizes (i.e., 3–5 amino acid residues) of *C*-locked analogs combined with fatty acids of different lengths (i.e., C_10_–C_14_) were investigated. The antimicrobial activity of the analogs was tested against *Staphylococcus aureus*, *Escherichia coli, Klebsiella pneumoniae*, *Acinetobacter baumannii*, and *Pseudomonas aeruginosa*. The most promising compound was analog **13** (MIC = 4 µg/mL (2.4 µM) against *E. coli* and 36% hemolysis of red blood cells at 150 µM). In a time-kill assay, this peptide showed a significant, concentration-dependent reduction in viable *E. coli* cells comparable to that seen for colistin.

## 1. Introduction

Multidrug-resistant bacteria that are unsusceptible to most or all antibiotics are becoming an increasing global health problem [1]. Especially critical bacteria comprise *Enterococcus faecium*, *Staphylococcus aureus*, *Klebsiella pneumoniae*, *Acinetobacter baumannii*, *Pseudomonas aeruginosa*, and Enterobacter species, which are referred to as the ESKAPE pathogens [2]. Since 2000, 38 new antibacterial agents have been marketed, of which five were first-in class, but these only have activity against Gram-positive bacteria [3]. Therefore, the development of antibiotics targeting Gram-negative bacteria is crucial in order to maintain efficacy in clinical therapy.

Antimicrobial peptides (AMPs) are a part of the innate immune system of all higher organisms [4]. Over the last decade, AMPs have attracted considerable interest as potential antibiotics [5]. Typical AMPs possess both cationic and hydrophobic moieties, which facilitate their membrane-specific mechanism of action. However, native AMPs have short half-lives and a low bioavailability, which may be circumvented by chemical modification such as cyclization and/or incorporation of non-standard amino acids (to provide more stable peptidomimetics) [6]. Despite these drawbacks, a number of AMPs are currently in clinical trials, albeit mainly for topical applications [5].

Cyclic lipopeptides (CLPs) belonging to the polymyxin family are currently considered last-resort antibiotics against infections caused by multidrug-resistant Gram-negative bacteria [7], while daptomycin is used for the treatment of infections caused by Gram-positive bacteria [8]. However, these compounds are known for their nephrotoxicity and neurotoxicity (polymyxins [9]) and hepatoxicity and nephrotoxicity (daptomycin [10,11]). The success of the CLPs has, however, renewed the interest in other CLP analogs as potential lead compounds against Gram-negative bacteria [12]. Unfortunately, resistance against CLPs is on the rise. For polymyxins, resistance is associated with an increased positive charge of the LPS layer of the Gram-negative outer membrane [13]. For Gram-positive bacteria, the most common view of daptomycin resistance is an increased positive charge of the peptidoglycan layer, while redistribution of acidic phospholipids at the septum has also been suggested [8].

Previously, Oddo et al. [14] reported a strategy for the on-resin synthesis of macrocyclic peptides via an intramolecular halide S_N_2 substitution involving a diamino acid such as lysine. The resulting macrocyclic peptide displays an endocyclic secondary amine allowing for the continued synthesis of a tail moiety. This chemistry facilitates previously disfavored high-throughput approaches for the design and screening of synthetic antimicrobial CLPs. BP214 is an cationic amphipathic all-D undecapeptide with an excellent activity against colistin-resistant *Acinetobacter baumannii* and modest hemolytic properties [15]. BP214 was developed from a short cecropin A-melittin hybrid peptide BP100 [16]. Our hypothesis is that combining structural elements from α-helices and cyclic lipopeptides may afford potent antimicrobials. In the present work, this is explored in a case study of the α-helical AMP BP214 by constraining a few of its amino acids into a macrocycle, as well as by incorporating a lipid tail. Here, we report the scope and limitations of this approach.

## 2. Results and Discussion

### 2.1. Synthesis

**Figure 1:**
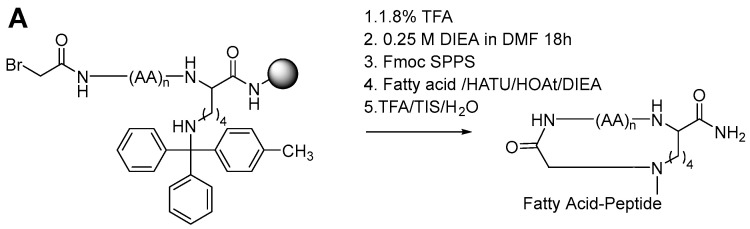

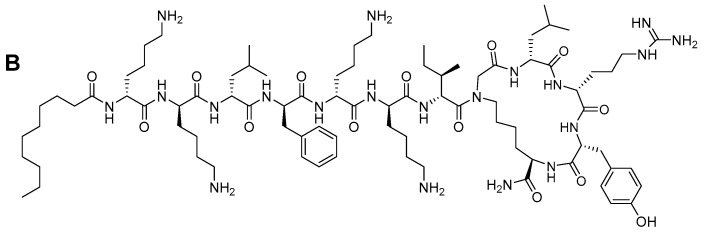
(**A**) General scheme for the synthesis of *C*-locked peptides. (**B**) An example is the lipidated *C*-locked BP214 analog **13**.

We synthesized 18 analogs of BP214 (Table 1): (i) six linear peptides carrying a fatty acid at the *N*-terminus, (ii) three *C*-locked analogs, and (iii) nine *C*-locked analogs also displaying an exocyclic lipidated peptide tail moiety (Figure 1A). The lipidated BP214 analogs were synthesized by using standard Fmoc-based solid-phase peptide synthesis (SPPS). SPPS of the *C*-locked BP214 analogs involves an initial construction of a linear intermediate with bromoacetyl group at the *N*-terminus and a momomethyltrityl (Mtt)-protected Lys residue attached at the *C*-terminus. The ε-amino Mtt protecting group was removed by repeated short treatments with 1.8% trifluoroacetic acid (TFA) in dichloromethane (DCM). Following macrocyclization overnight, which generates a secondary amine, the lipopeptide exocyclic chain was then assembled by standard Fmoc-based SPPS. All 18 peptide analogs were screened for antimicrobial activity against *Staphylocuccos aureus*, *Escherichia coli*, *Klebsiella pneumoniae*, *Acinetobacter baumannii*, and *Pseudomonas aeruginosa*, and their hemolytic activity against human erythrocytes was also determined. BP214 was included for comparison. The peptide sequences, MIC values, and hemolytic activity are shown in Table 1.

#### 2.1.1. Fatty Acid Conjugates of BP214, Compounds **1**–**6**

As the lipidation of AMPs may have a significant effect on activity [17], we first investigated the effect of conjugating butanoic, hexanoic, octanoic, decanoic, lauric and myristic acids to BP214. The resulting C_4_, C_6_, and C_8_ lipidated BP214 analogs (i.e., **1**–**3**) showed a good activity (2–16 μg/mL) against the Gram-negative bacteria *E. coli*, *K. pneumoniae*, and *A. baumanii*, and a moderate activity against *P. aeruginosa* (32 μg/mL). However, these analogs were not active against *S. aureus* (64 μg/mL). 

We observed the analogs **1**–**3** (C4, C6, and C8) to be effective against Gram-negative, but not against the Gram-positive *S. aureus*. This is probably because the envelope of Gram-negative bacteria is very different from that of Gram-positive bacteria. In the latter, the envelope consists of a cytoplasmic membrane surrounded by a thick layer of peptidoglycan. In contrast, the Gram-negative envelope consists of a cytoplasmic membrane, a thin layer of peptidoglycan, and an outer membrane. The outer membrane is an asymmetric bilayer of phospholipid in the inner leaflet and primarily lipopolysaccharide molecules in the outer leaflet [18]. LPS molecules contain numerous negatively charged phosphate groups involved in salt bridges with divalent cations [19]. We speculate that the analogs displace the divalent cations in a mechanism similar to that of colistin [20].

The C_10_, C_12_, and C_14_ analogs (i.e., **4**–**6**) showed a significantly reduced antimicrobial activity as compared to that of BP214 (i.e., 32 μg/mL, 64 μg/mL, and >64 μg/mL). This is in accordance with Chen et al., who found that hydrophobic compounds may possess a reduced antimicrobial activity due to more pronounced intermolecular hydrophobic interactions, thereby leading to aggregation of the peptides [21]. As expected, all lipidated linear analogs were proven to be very hemolytic, which is highly unfavourable for a potential drug candidate. Thus, lipidation of BP214 neither improved the antimicrobial activity nor lowered the hemolytic properties of BP214, and none of these six analogs were considered further.

#### 2.1.2. Synthesis of *C*-Locked Analogs of BP214, Compounds **7**–**9**

Cyclization of AMPs often improves their antimicrobial activity and reduce their toxicity more effectively than any substitution of residues [22]. Thus, three BP214 analogs with a cyclic moiety at the C-terminus, here termed *C*-locked analogs, were synthesized. These BP214 analogs were varied with respect to ring size (i.e., 3–5 amino acid residues) to provide analogs **7**–**9**, which also were tested for their antimicrobial and hemolytic activity. Their synthesis is shown in Figure 1A. All three *C*-locked analogs **7**–**9** exerted a significantly reduced antimicrobial activity (i.e., MIC > 64 μg/mL). This is in accordance with Tamaki et al., who reported similar results for cyclized AMPs (with rings consisting of 6–9 amino acids), which also showed a weak antimicrobial activity [23]. Interestingly, all three analogs exhibited significantly reduced hemolytic properties. As no other chemical modifications were made in the three analogs, it was expected for the hemolytic activity to be unchanged or better. The hypothesis was supported by the results published by Kondejawski et al. [24]. They found that small rings (4–8 amino acid residues) lowered the hemolytic activity of compounds as compared with the corresponding linear templates. Thus, macrocyclization reduced the already low hemolytic activity of BP214 further, but unfortunately also essentially abolished the antimicrobial activity. None of these three analogs were considered further.

#### 2.1.3. Synthesis of Lipidated *C*-Locked Analogs of BP214, Compounds **10**–**18**

We then synthesized nine *C*-locked analogs containing both macrocyclic and fatty acid moieties. *C*-locked analogs (with a ring size of 3–5 amino acid residues) carrying C_10_ to C_14_ fatty acids were investigated. For the C3-ring series (i.e., **10**–**12**), we found no significant changes in MIC values compared with those of BP214, except for the C_14_ analog **12**, which showed a decreased activity (MIC of 16 μg/mL) against *A. baumannii* and *P. aeruginosa*. For the C4-ring series (i.e., **13**–**15**), the only significant difference was seen against *K. pneumoniae*, where all three lipidated analogs showed a reduced potency (i.e., a MIC of 16 μg/mL). Similarly, within the C5-ring series (i.e., **16**–**18**), all three lipidated analogs had an activity similar to that of BP214, except for *K. pneumoniae* which showed a reduced susceptibility (i.e., MICs of 16–64 μg/mL). 

When comparing the different ring sizes combined with the presence of the same fatty acid, we found that all C_10_ analogs (i.e., **10**, **13**, and **16**) had similar MIC values compared with those of BP214—except for the lower potency against *K. pneumoniae* (i.e., MICs of 8–16 μg/mL). This was also the case for the *C*-locked analogs carrying C_12_ (i.e., **11**, **14**, and **17**) or C_14_ (i.e., **12**, **15**, and **18**) fatty acids, where only **12** retained comparable potency against *K. pneumoniae*, whereas other representatives of these subseries displayed a lower activity (with MICs in the range of 8–64 μg/mL).

Expectedly, the length of the fatty acid affected the hemolytic activity of the analogs. All analogs carrying C_12_ (i.e., **11**, **14** and **17**) or C_14_ (i.e., **12**, **15** and **18**) fatty acids exhibited 83% or higher hemolysis against erythrocytes (at 150 μM), which is unacceptable for compounds intended for systemic administration, and therefore the C_12_ and C_14_ analogs were not considered further. Alternatively, these compounds may be relevant for topical applications. This strategy has been applied for several other AMPs in the clinical pipeline, e.g., pexiganan and lytixar [25]. The ring size was the only difference between the C_10_ analogs **10**, **13**, and **16**, which exhibited 81%, 36%, and 59% hemolysis, respectively. Therefore, we speculate that the difference in hemolytic properties in part may be related to ring constraints. The three-membered ring is highly constrained with a less flexible lysine side chain compared with the four- and five-membered structures (see Supplementary Materials for structures). Importantly, analogs **13** and **16** showed a comparable antimicrobial activity to that of BP214. 

However, analog **13** was chosen as the most promising peptide analogue, since it was the only compound that had a similar hemolytic activity compared to BP214. The hemolysis concentration–response curve of BP214, analog **13**, and analog **8** (ring without the fatty acid chain) are shown in the Supplementary Materials. It can be seen that BP214 is more hemolytic than analog **13** at lower concentrations. Both compounds have a hemolytic activity of ca. 40% at 150 μM, which is unacceptable for systemic use. Thus, even though cyclization and a fatty acid moiety by themselves did not improve the antimicrobial activity of BP214, the combination of these two structural alterations led to a retained antimicrobial activity. When MIC values and hemolytic activity were considered together, analogue **13** was found to be the best candidate for further investigation. 

#### 2.1.4. Time-Kill Assay of Analog **13**

**Figure 2:**
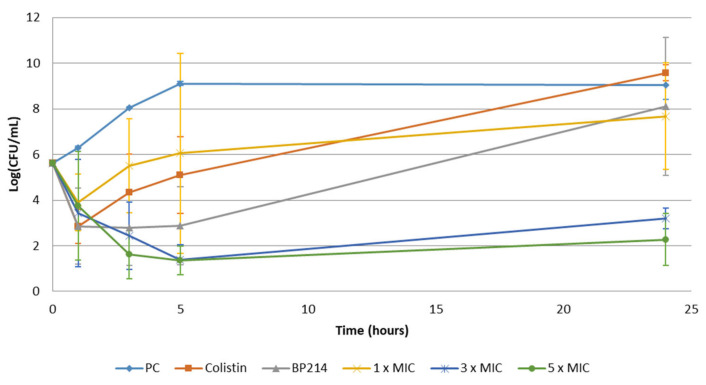
Time-kill kinetics of analogue **13** against *E. coli*. The following compounds were tested: Colistin; 1 × MIC, BP214; 1 × MIC, and **13**; 1 × MIC, 3 × MIC and 5 × MIC. In addition, a positive control (PC) was included. Samples were collected at time points = 0, 1, 3, 5, and 24 h (n = 3, mean × standard deviation) post treatment.

To achieve an understanding of the killing kinetics of analog **13**, a time-kill assay was performed on *E. coli* cells. Three different concentrations of analog **13**, namely 1 × MIC (4 μg/mL), 3 × MIC (12 μg/mL), and 5 × MIC (20 μg/mL), were employed in the experiment. For comparison, 1 × MIC of colistin (0.25 μg/mL) and 1 × MIC of BP214 (4 μg/mL) were included. The limit of detection was set to 10 CFU/mL, corresponding to a single viable colony after the overnight incubation of 100 μL bacteria suspension on an agar plate. A graphic illustration of the results obtained from the time-kill assay (performed in triplicate) is shown in Figure 2.

The results obtained with both 3 × MIC and 5 × MIC of analogue **13** showed a prolonged killing over 5 h compared with that seen with 1 × MIC. After 24 h, the number of viable cells was reduced from 5 × 10^5^ CFU/mL to 10^3^ CFU/mL and 10^2^ CFU/mL for 3 × MIC and 5 × MIC, respectively. The two concentrations gave similar results, suggesting that both concentrations provide an excess amount of drug molecules needed to kill the majority of bacterial cells. This is similar to what was previously observed for BP214 at 2 × MIC, 4 × MIC, and 8 × MIC [15]. At 4 × MIC and above, BP214 was able to reduce the number of CFU of the colistin-susceptible strain *A. baumannii* ATCC 19606 to below the detection level. 

The rapid reduction in CFU numbers over time was similar to what was observed for other amphipathic peptides [26,27], and it is highly indicative of a mode of action that involves the loss of membrane integrity. An increase in viable cells over time was observed for all concentrations of analog **13**. This could result from the appearance and growth of resistant bacteria. This was, however, not tested. Alternatively, regrowth could result from partial killing due to compound sequestration. If the latter is the case, the use of analog **13** may still be relevant in an in vivo setting, as a significant reduction in viable bacterial cells might be sufficient for the immune system to kill the remaining cells [28]. Overall, AMP analog **13** displays concentration-dependent killing with an *E. coli* in vitro killing profile similar to that of both colistin and BP214. The majority of *E. coli* cells were killed within the first hour after application, with an efficacy of approximately 97%.

## 3. Materials and Methods

### 3.1. Chemicals

TFA was from Alfa Aesar (Haverhill, MA, USA); ACCA from Bruker (Ettlingen, Germany); butanoic acid, hexanoic acid, octanoic acid, and decanoic acid (C_10_) were from Fluka Analytical (Buchs, Switzerland); and HOAt and HATU were from GLS Biochem (Shanghai, China). DTT, Fmoc-D-Arg(Pbf)-OH, Fmoc-D-Ile-OH, Fmoc-D-Lys(Boc)-OH, Fmoc-D-Lys(Mtt)-OH, Fmoc-D-Phe-OH, Fmoc-D-Tyr(tBu)-OH, piperidine, and TentaGel^®^ S RAM (loading 0.24 mmol/g) were from Iris Biotech GmbH (Marktredwitz, Germany). Bromoacetic acid (BrAcOH), lauric acid (C_12_), myristic acid (C_14_), DIC, DIEA, DMSO, Fmoc-D-Asn(Trt)-OH, and TIS were from Sigma-Aldrich (St. Louis, MO, USA). ACN, DCM, diethyl ether, DMF, ethanol, and methanol were from VWR International (Radnor, PA, USA).

### 3.2. Microbiology

The following bacteria were grown in cation-adjusted Muller-Hinton broth at 37 °C with shaking: *A. baumannii* (ATCC 19606), *E. coli* (ATCC 25922), *K. pneumoniae* (ATCC 13883), *P. aeruginosa* (ATCC 27853), and *S. aureus* (ATCC 29213).

### 3.3. Hemolysis

Melittin (Carbosynth, Staad, Switzerland), PBS (Sigma-Aldrich, St. Louis, MO, USA), Fresh type 0 neg whole blood in citrate 96-well polypropylene plates with conical bottom, 96-well clear, polystyrene ELISA plates with flat bottom. CAPPOrigami reagent reservoirs (VWR International, Radnor, PA, USA), Microseal™ ‘F’foil (Bio-Rad, Hercules, CA, USA), Protein LoBind Eppendorf tubes, 2 mL (Eppendorf, Hamburg, Germany). Molecular Devices VersaMax^TM^ Tunable Microplate Reader (San Jose, CA, USA), Holm and Halby plate centrifuge B 4i (Copenhagen, Denmark).

### 3.4. Peptide Synthesis

#### 3.4.1. Synthesis of Lipidated BP214 Conjugates

Linear peptides were synthesized manually by Fmoc-based SPPS on a Tentagel S RAM resin (loading 0.2 mmol/g) in 5 mL polypropylene reactors fitted with a PTFE filter. The synthesis scale was 0.02 mmol. After swelling overnight in DMF, the resin was washed thoroughly with DMF. Peptide bond formation was done by using Fmoc-protected amino acid building blocks/HATU/HOAt/DIEA (5:5:5:10 equiv) in DMF for 2 h. Deprotection of the Fmoc group was achieved by using 20% piperidine in DMF for 4 × 3 min. For the exocyclic chain, the deprotection times were increased (2 × 5 min and 1 × 7 min). The resin was washed with DMF (3×), DCM (3×), and DMF again (5×) before and after the piperidine treatment. Fatty acids were coupled as described for the amino acids. The resin was then washed with 3 × DMF, 3 × DCM, and 5 × EtOH and freeze-dried overnight.

#### 3.4.2. Cleavage from Resin

Lipidated BP214 conjugates were cleaved from the resin by using TFA:DTT:H_2_O:TIS (88:5:5:2) for 4 h. Initially, we used TFA:H_2_O:TIS (95:2.5:2.5) for 2 h; however, we noticed that the Pbf group on arginine was not completely deprotected under these conditions. Following evaporation of the cleavage cocktail, the crude product was precipitated in ether, redisolved in ACN:H_2_O (1:1), and freeze-dried. 

#### 3.4.3. Synthesis of *C*-Locked BP214 Analogs and Lipidated *C*-Locked BP214 Analogs

Starting with a resin-bound Lys(Mtt) as the C-terminal amino acid residue, the linear peptide chain was synthesized as described above. After Fmoc deprotection of the third, fourth, or fifth residue, BrAcOH (20 equiv) and DIC (20 equiv) were pre-activated for 3 min in DMF and then coupled to the resin for 25 min. The resin was then washed with 3 × DMF and 3 × DCM, and then treated repeatedly with 1.8% TFA in DCM (2 mL; each time for 3 min) until the color of the liquid changed from yellow to a dark orange for the removal of the Mtt group. Prior to cyclization, the resin was washed with 1 × DCM, 3 × 5% DIEA in DCM, and 5 × DMF. Then, 0.25 M DIEA in DMF was transferred to the syringe and placed on a shaker overnight. The exocyclic chain was then synthesized by Fmoc-based SPPS, followed by lipidation with the appropriate fatty acid, and finally cleaved from the resin as described above. For *C*-locked BP214 analogs, the fatty acid acylation step was omitted. 

#### 3.4.4. RP-HPLC and MALDI-TOF-MS

Peptide purity was determined as described previously [29] by analytical RP-HPLC using a Waters 717 plus Autosampler, In-line Degasser AF, 600 Controller, and 2996 Photodiode Array Detector; the column used was a Waters Symmetry C18 column (5 μm, 4.6 mm × 250 mm, Milford, MA, USA). An aqueous acetonitrile (ACN) gradient with 0.1% TFA added (mixing eluent A: H_2_O + 0.1% TFA and eluent B: 90:10 ACN-H_2_O + 0.1% TFA) was employed using milli-Q water. Preparative RP-HPLC was performed by using a Waters XSelect Peptide CSH C18 OBDTM column (5 μm, 19 mm × 250 mm, Milford, MA, USA) with the same eluents as for analytical HPLC. 

Microflex^TM^ (Bruker Corporation, Bremen, Germany) equipped with FlexControl software (Bruker Daltonik GmbH, Bremen, Germany) was used to obtain the MALDI-TOF-MS spectra, and the data were processed using flexAnalysis (Bruker Daltonik GmbH). All of the reagents and solvents were used without further purification.

### 3.5. Hemolysis

The hemolytic effect of each peptide, including BP214, was measured according to Oddo and Hansen [30] at concentrations ranging from 2.35 μM to 150 μM. PBS served as the negative control and 5 μM melittin served as the positive control. All melittin solutions were prepared in Protein LoBind Eppendorf tubes to prevent the adsorption of melittin to the inner surface of the tubes.

Preparation of peptide solutions and melittin: A 5 μM melittin solution in PBS was prepared the day before the hemolysis experiment. First, 150 μL of this solution was transferred to each of the positive control wells and stored in the fume hood to coat the wells. On the day of the experiment, the melittin solution was discarded and the wells were washed with 3 × PBS. A 2.5 μM melittin solution in PBS was prepared and 75 μL was transferred to the wells. PBS (150 μL) was used as the negative control. A solution of each peptide was prepared by dissolving the peptide in 0.5 mL PBS to obtain a concentration of 300 μM. Each peptide solution (150 μL) was transferred to the wells in three wells each in row A, and the plate was diluted down. 

Preparation of the red blood cell suspension: 1 mL of type O- whole blood in citrate was transferred to a cryotube. The whole blood was washed with 3 × PBS; adding 3, 3, and 4 mL of PBS, respectively. In between, the blood was centrifuged for 8 min (3000, 3000, and 4000 rpm, respectively) and the supernatant was removed after each centrifugation. A 0.5% (*v/v*) blood suspension was prepared by adding 40 μL of the washed blood cells to 8 mL of PBS. Then, 75 μL of the red blood cell suspension was transferred to each well and mixed gently. The plate was sealed and incubated for 1 h at 37 °C. After incubation, the plate was centrifuged at 4000 rpm for 10 min. Afterwards, 60 μL of the supernatant from each well was quickly transferred to an ELISA plate.

The absorbance was determined at 414 nm, and Equation (1) below was used to calculate the hemolytic activity of the peptides.

(1) $$\%\;\mathrm{Hemolysis}=\left(\frac{\mathrm{Apeptide}-\mathrm{Aneg}\left(\mathrm{mean}\right)}{\mathrm{Apos}\left(\mathrm{mean}\right)-\mathrm{Aneg}\left(\mathrm{mean}\right)}\right)\times 100$$

### 3.6. Antimicrobial Activity

The MIC experiments were performed in triplicate, as described in Jensen et al. [29]. Briefly, all peptide stock solutions were prepared by dissolving each peptide in Milli-Q water to obtain a concentration of 10 mg/mL. A bacterial concentration of 10^6^ CFU/mL was obtained through the dilution of an overnight culture (MHB-II medium). When the overnight culture was prepared, the OD was measured using a spectrophotometer and when an OD value between 0.2 and 0.4 was obtained, the culture was diluted to OD = 0.002. The MIC determination was carried out in 96-well polypropylene microtitre plates. BP214 analogs were prepared as two-fold dilutions in 50 μL volumes. A final bacterial inoculum of 5 × 10^5^ CFU/mL per well was obtained by adding 50 μL bacterial suspension to the microtiter plates. The plates were incubated at 37 °C for 18–24 h. To verify the CFU, 10 μL of bacteria suspension was transferred to 990 μL 0.9 % saline solution, from which a 10-fold dilution was carried out. Then, 100 μL of the dilution was plated on an LB plate and left for bacterial growth overnight at 37 °C. Colistin and vancomycin were used as the positive controls.

### 3.7. Time-Kill Assay for the Most Promising Peptide Candidate

An overnight culture (MHB-II media) of *E. coli* was diluted 1:100 in preheated MHB-II, placed in a water bath (37 °C), and allowed to grow until OD_600_ = 0.2–0.4 was reached. The bacterial suspension was then diluted 1:10 in 25 mL preheated MHB-II, placed in a water bath (37 °C), and then allowed to grow until an OD = 0.2 was reached. Then, the suspension was diluted into several flasks to obtain OD = 0.001 (≈5 × 10^5^ CFU/mL) in preheated medium, containing analog 13 (1, 3, and 5 × MIC), colistin (1 × MIC), or BP214 (1 × MIC). At time points 0, 1, 3, 5, and 24 h, 250 μL of each sample suspension were transferred to a 0.9 % saline solution in a 1-mL Eppendorf tube. The bacterial cells were washed twice in 0.9% saline, spun down (10,000× g for 5 min) and resuspended in 250 μL 0.9% saline. Then, 10-fold dilution series were prepared and aliquots of 10 μL were spotted onto LB Agar. In addition, 100 μL of undiluted bacterial suspension (except the control sample) was plated out on a separate LB plate. All of the plates were incubated (37 °C) overnight and the results were read after incubation.

## 4. Conclusions 

In the present work, we explored a strategy for the synthesis of cyclic lipopeptides, termed *C*-locked analogs, involving a combination of Fmoc-based SPPS and the submonomer approach. Macrocyclization was achieved by reacting the ε-amino group on the C-terminal lysine residue with a bromoacetyl group attached to the *N*-terminus of a resin-bound intermediate. The resulting secondary amine serves as an anchoring point for the assembly of an exocyclic lipidated peptide tail via standard SPPS, which constitutes an efficient strategy for obtaining CLPs in good yields with a satisfactory crude purity.

Our best candidate was analog **13**, which exhibited a comparable antimicrobial and hemolytic activity to BP214. The current work is directed at investigating the properties of this compound and the synthesis of *N*-locked analogs.

## Figures and Tables

**Table 1:** Minimum inhibitory concentration values in μg/mL(μM) and % hemolysis against human erythrocytes at 150 μM.

ID	Peptide	SA ^a^	EC ^b^	KP ^c^	AB ^d^	PA ^e^	%H ^f^
BP214	kklfkkilryl-NH_2_	8(5.5)	4(2.8)	2(1.4)	2(1.4)	4(2.8)	41
**1**	C_4_-kklfkkilryl-NH_2_	64(42.1)	2(1.3)	4(2.6)	2(1.3)	32(21.1)	100
**2**	C_6_-kklfkkilryl-NH_2_	64(41.4)	8(5.2)	8(5.2)	4(2.6)	32(20.7)	100
**3**	C_8_-kklfkkilryl-NH_2_	64(40.1)	16(10.2)	8(5.1)	4(2.5)	32(20.3)	100
**4**	C_10_-kklfkkilryl-NH_2_	32(20.0)	32(20.0)	32(20.0)	32(20.0)	32(20.0)	100
**5**	C_12_-kklfkkilryl-NH_2_	64(39.2)	64(39.2)	64(39.2)	64(39.2)	64(39.2)	100
**6**	C_14_-kklfkkilryl-NH_2_	>64(38.6)	>64(38.6)	>64(38.6)	>64(38.6)	>64(38.6)	100
**7**	kklfkkil**ryk** ^g^-NH_2_	>64(42.6)	>64(42.6)	>64(42.6)	>64(42.6)	>64(42.6)	3
**8**	kklfkki**lryk**-NH_2_	>64(42.6)	>64(42.6)	>64(42.6)	>64(42.6)	>64(42.6)	3
**9**	kklfkk**ilryk**-NH_2_	>64(42.6)	>64(42.6)	>64(42.6)	>64(42.6)	>64(42.6)	3
**10**	C_10_-kklfkkil**ryk**-NH_2_	4(2.4)	4(2.4)	8(4.8)	4(2.4)	4(2.4)	81
**11**	C_12_-kklfkkil**ryk**-NH_2_	4(2.3)	4(2.3)	8(4.6)	4(2.3)	4(2.3)	96
**12**	C_14_-kklfkkil**ryk**-NH_2_	8(4.6)	8(4.6)	4(2.3)	16(9.3)	16(9.3)	95
**13**	C_10_-kklfkki**lryk**-NH_2_	8(4.8)	4(2.4)	16(9.6)	2(1.2)	8(4.8)	36
**14**	C_12_-kklfkki**lryk**-NH_2_	4(2.4)	4(2.4)	16(9.5)	4(2.4)	4(2.4)	98
**15**	C_14_-kklfkki**lryk**-NH_2_	4(2.3)	4(2.3)	16(9.3)	4(2.3)	8(4.6)	83
**16**	C_10_-kklfkk**ilryk**-NH_2_	4(2.4)	8(4.8)	16(9.7)	4(2.4)	8(4.8)	59
**17**	C_12_-kklfkk**ilryk**-NH_2_	4(2.4)	8(4.7)	64(40.0)	4(2.4)	8(4.8)	100
**18**	C_14_-kklfkk**ilryk**-NH_2_	4(2.3)	8(4.6)	32(18.7)	4(2.3)	16(9.3)	94
**19**	Colistin	N/A	0.25(0.2)	0.5(0.4)	0.25(0.2)	0.5(0.4)	N/A
**20**	Vancomycin	0.5(0.35)	N/A	N/A	N/A	N/A	N/A

^a^*S. aureus* (ATCC 29213), ^b^ *E. coli* (ATCC 25922), ^c^ *K. pneumoniae* (ATCC 13883), ^d^ *A. baumannii* (ATCC 19606), ^e^ *P. aeruginosa* (ATCC 27853), ^f^ % hemolysis of human erythrocytes at 150 μM. ^g^ Residues in bold are part of the *C*-locked ring structure. N/A: not applicable. Melittin (5 μM) was used as a reference in hemolysis experiments.

## Data Availability

The data presented in this study are available in Supplementary Material.

## Supplementary Materials

The following are available online at https://www.mdpi.com/article/10.3390/antibiotics11081080/s1, SI: Structure of BP214 analogs; SII: Overview of the analytical data obtained by MALDI-TOF-MS and RP-HPLC; SIII: Analytical chromatograms after purification SIV: MALDI-TOF-MS spectra of all purified peptides; SV: Hemolysis dose response curves for BP214, [C4]BP214 and [(C10)C4]BP214.

## Abbreviations

ACCA	α-cyano-4-hydroxycinnamic acid
ACN	Acetonitrile
AMP	Antimicrobial peptide
ATCC	The American Type Culture Collection
BrAcOH	Bromoacetic acid
CFU	Colony-forming units
DCM	Dichloromethane
DIEA	Diisopropylethylamine
DMF	Dimethylformamide
DTT	Dithiothreitol
ELISA	Enzyme-linked immunosorbent assay
Fmoc	9-fluorenylmethoxycarbonyl
HATU	1-[Bis(dimethylamino)methylene]-1*H*-1,2,3-triazolo[4,5-b] pyridinium-3-oxide hexafluoro-phosphate
HOAt	1-Hydroxy-7-azabenzotriazole
MALDI-TOF-MS	Matrix-assisted Laser Desorption-Ionization Time-of-Flight Mass Spectrometry
MHB	Mueller-Hinton Broth
Mtt	4-methyltrityl
OD	Optical density
Pbf	2,2,4,6,7-pentamethyldihydrobenzofuran-5-sulfonyl
PBS	Phosphate-buffered saline
RAM	Rink amide Linker
RP-HPLC	Reverse Phase Analytical High Performance Liquid Chromatography
TFA	trifluoroacetic acid
TIS	Triisopropylamine
