# Supplementary material for: C-Locked Analogs of the Antimicrobial Peptide BP214
Source: Antibiotics (Basel). 2022 Aug 9;11(8):1080. doi: 10.3390/antibiotics11081080 (PMC9404711; doi:10.3390/antibiotics11081080)
Supplement: Supplementary file 1 [file antibiotics-11-01080-s001.zip › antibiotics-1819589-supplementary.pdf]

Supporting Information

C-locked analogs of the antimicrobial peptide BP214

Ida Kristine Lysgaard Andersen [1,2], Thomas T. Thomsen [3,4,5], Jasmina Rashid[1], Thomas Rønnemos Bobak[1], Alberto Oddo[1,6], Henrik Franzyk[1], Anders Løbner-Olesen [4] and Paul R. Hansen[1,*]

[1] Department of Drug Design and Pharmacology, Faculty of Health and Medical Sciences, University of Copenhagen, Universitetsparken 2, 2100 Copenhagen, Denmark;

[2] Present Address: Department of Agroecology, University of Aarhus, Forsøgsvej 1, 4200 Slagelse, Denmark. ida.andersen@agro.au.dk.

[3] Department of Clinical Microbiology, Rigshospitalet, Henrik Harpestrengs Vej 4A, 2100 Copenhagen. Denmark

[4] Department of Biology, Section for functional Genomics, University of Copenhagen, Ole Maaløes Vej 5, 2200 Copenhagen, Denmark; lobner@bio.ku.dk

[5] Present Address: Sniper Biome, Lersø Parkallé 44, 2100 Copenhagen, Denmark. tt@sniprbiome.com

[6] Present Address: Novozymes; albi.oddo@gmail.com

Table of Contents:

SI: Structure of BP214 analogues

SII: Overview of the analytical data obtained by MALDI-TOF-MS and RP-HPLC

SIII: Analytical chromatograms after purification

SIV: MALDI-TOF-MS spectra of all purified peptides

SV: Hemolysis dose response curves for BP214, [C4]BP214 and [(C10)C4]BP214.

SI: Structure of BP214 analogs

R (in blue) below represents the following fatty acids:

C4 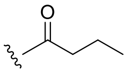 C6 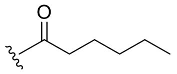 C8 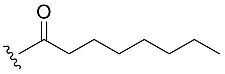

C10 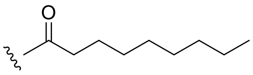 C12 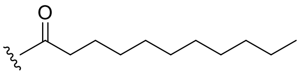 C14 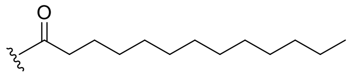

Compounds 1-6:

[C_4]BP214 (2), [C_6]BP214 (3), and [C_8]BP214 (4), [C_{10}]BP214 (5), [C_{12}]BP214 (6), and [C_{14}]BP214 (7).

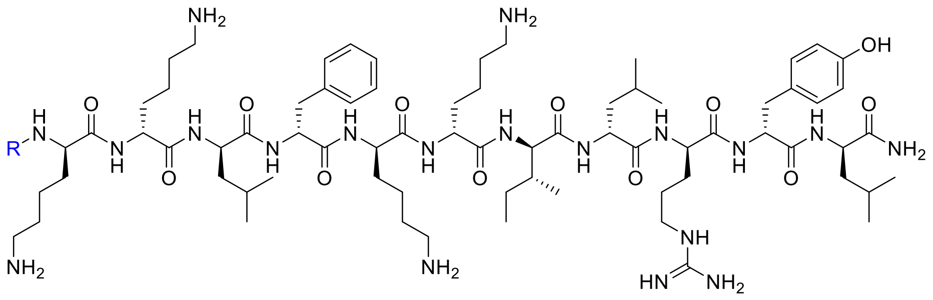

[*C*3]BP214 (7)

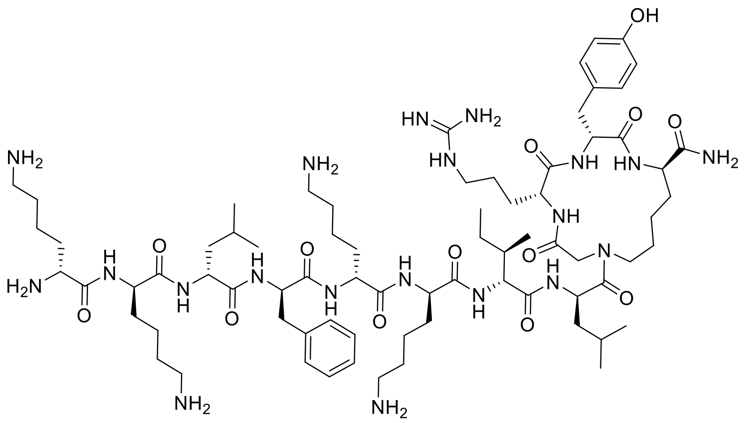

[*C*4]BP214 (8)

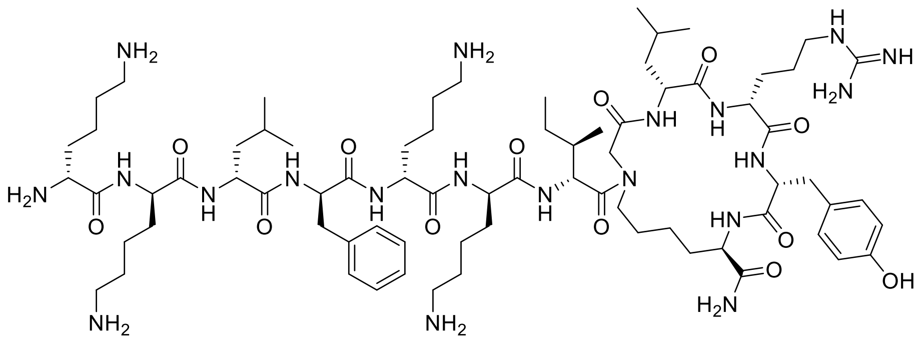

[*C*5]BP214 (9)

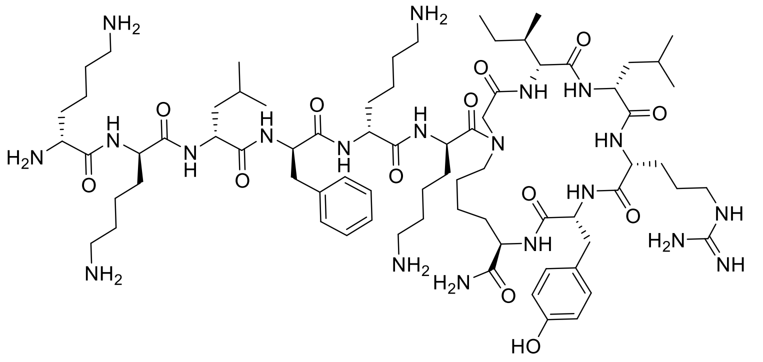

[(C_{10})*C*3]BP214 (10), [(C_{12})*C*3]BP214 (11), and [(C_{14})*C*3]BP214 (12)

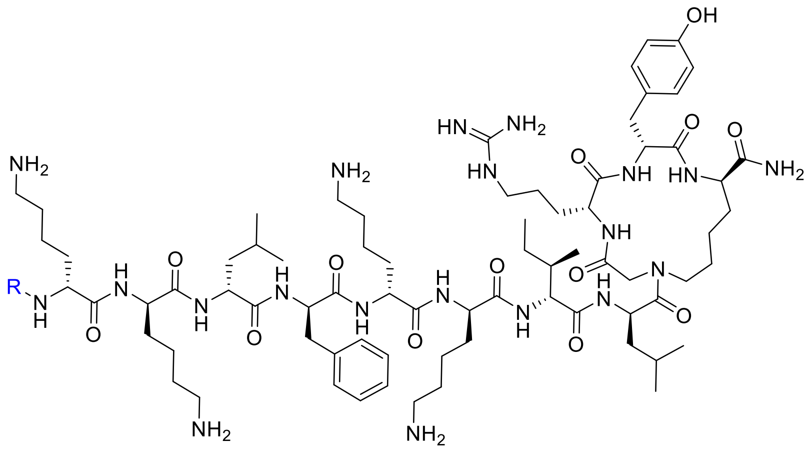

[(C_{10})*C*4]BP214 (13), [(C_{12})*C*4]BP214 (14), and [(C_{14})*C*4]BP214 (15).

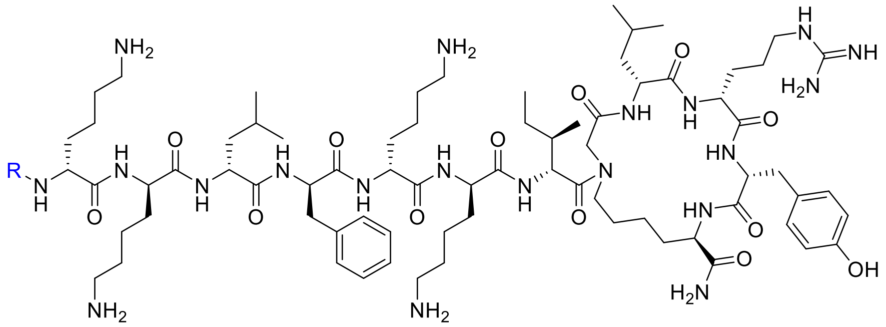

[(C10)*C*5]BP214 (16), [(C12)*C*5]BP214 (17), and [(C14)*C*5]BP214 (18).

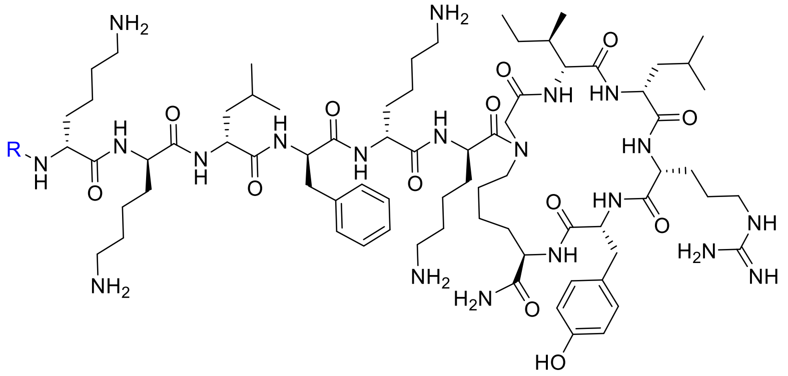

SII: Schematic overview of the synthesised BP214 analogs

Peptide name	Theoretical Mw (Da)	Yield (mg)	%B*	Purity (%)
BP214	1448.91	42.1	50.8	100.0
[C_4]BP214	1519.00	9.3	63.0	98.5
[C_6]BP214	1547.06	5.9	67.9	99.9
[C_8]BP214	1575.11	14.0	64.1	95.4
[C_{10}]BP214	1603.17	27.6	85.0	98.1
[C_{12}]BP214	1631.22	31.0	90.5	98.5
[C_{14}]BP214	1659.27	24.5	96.5	95.3
[*C3*]BP214	1503.95	2.2	44.1	99.7
[*C*4]BP214	1503.95	14.5	44.8	100.0
[*C*5]BP214	1503.95	6.5	46.6	99.5
[(C_{10})*C*3]BP214	1658.20	4.1	62.7	93.0
[(C_{12})*C*3]BP214	1686.26	6.2	66.2	99.4
[(C_{14})*C*3]BP214	1714.31	4.4	70.0	96.6
[(C_{10})*C*4]BP214	1658.20	16.1	60.9	97.8
[(C_{12})*C*4]BP214	1686.26	23.1	65.1	98.1
[(C_{14})*C*4]BP214	1714.31	22.5	78.5	99.5
[(C_{10})*C*5]BP214	1658.20	9.3	61.3	97.8
[(C_{12})*C*5]BP214	1686.26	15.1	65.5	93.9
[(C_{14})*C*5]BP214	1714.31	17.2	78.5	99.5

*Hydrophobicity as indicated by the percentage of mobile phase B (%B) at the peak elution time on analytical reversed-phase HPLC.

SIII:Analytical HPLC chromatograms after purification

BP214

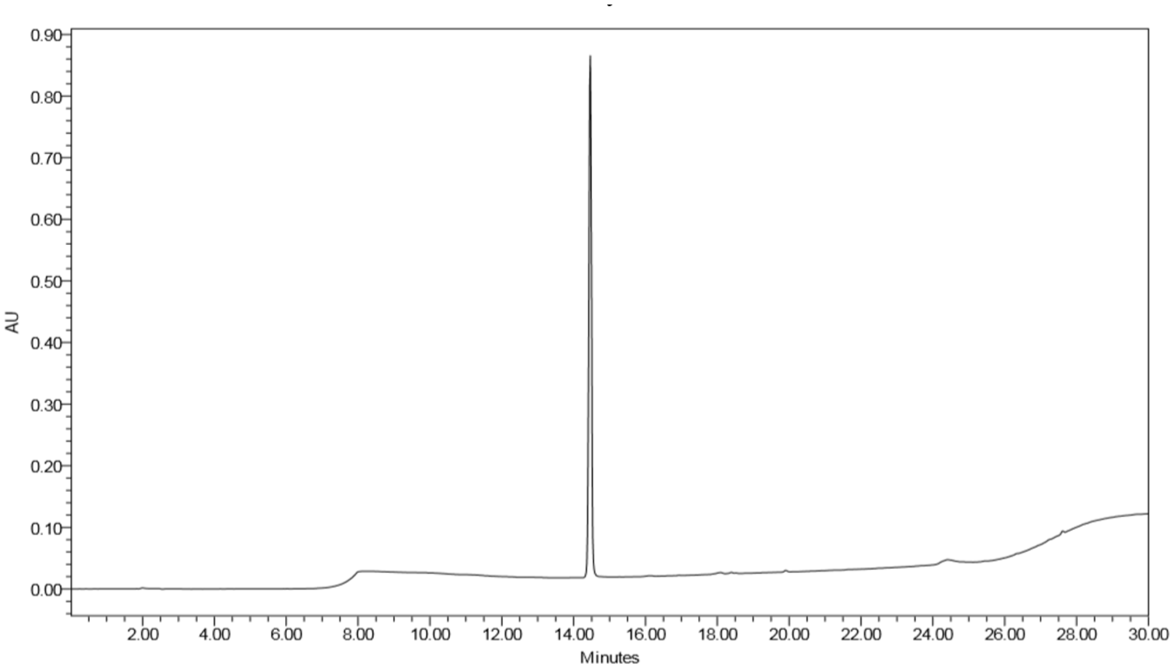

[C4]BP214 (**1**)

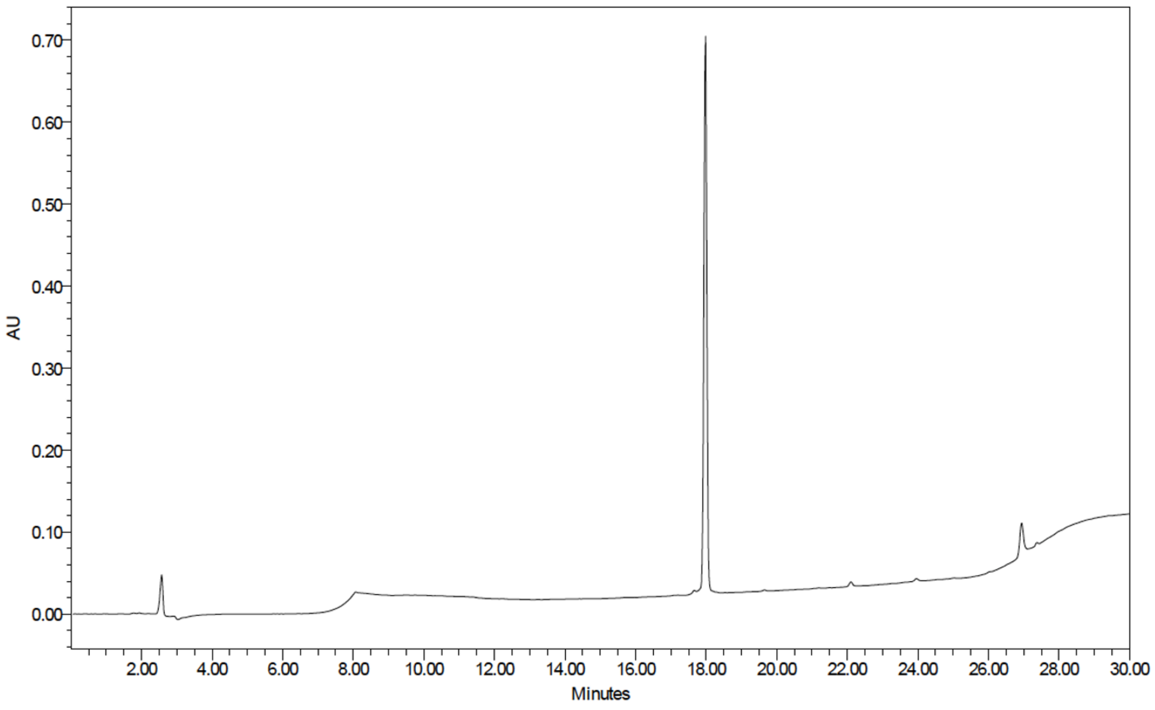

[C6]BP214 (**2**)

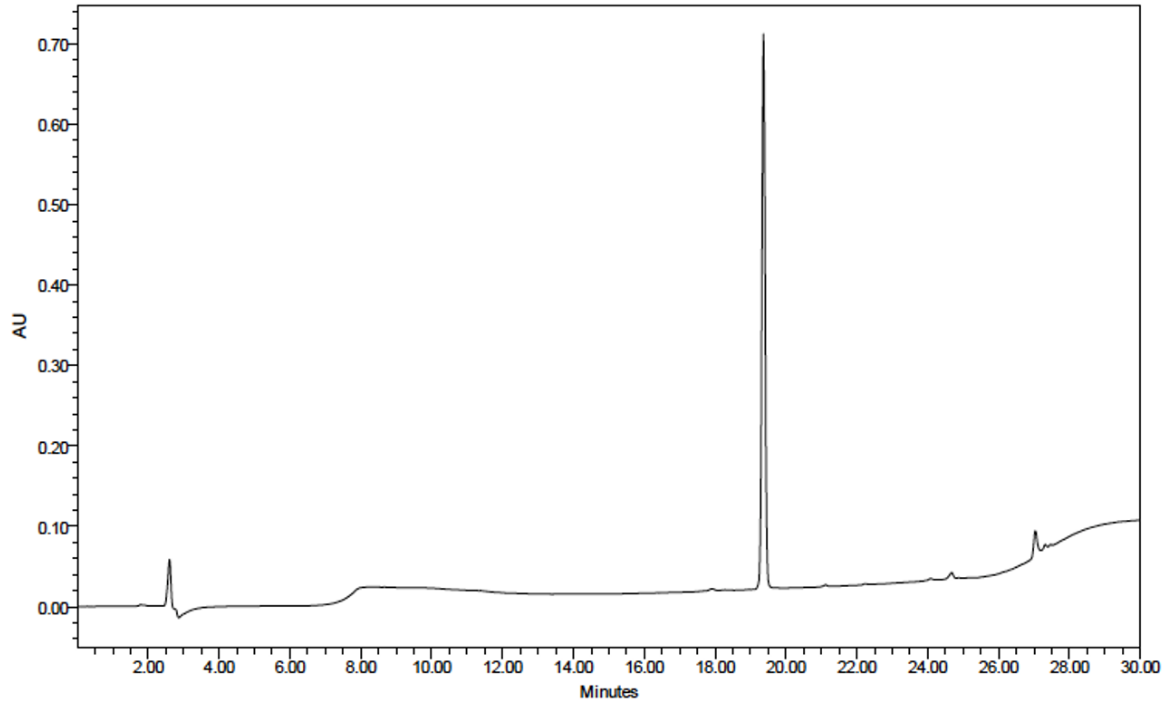

[C8]BP214 (**3**)

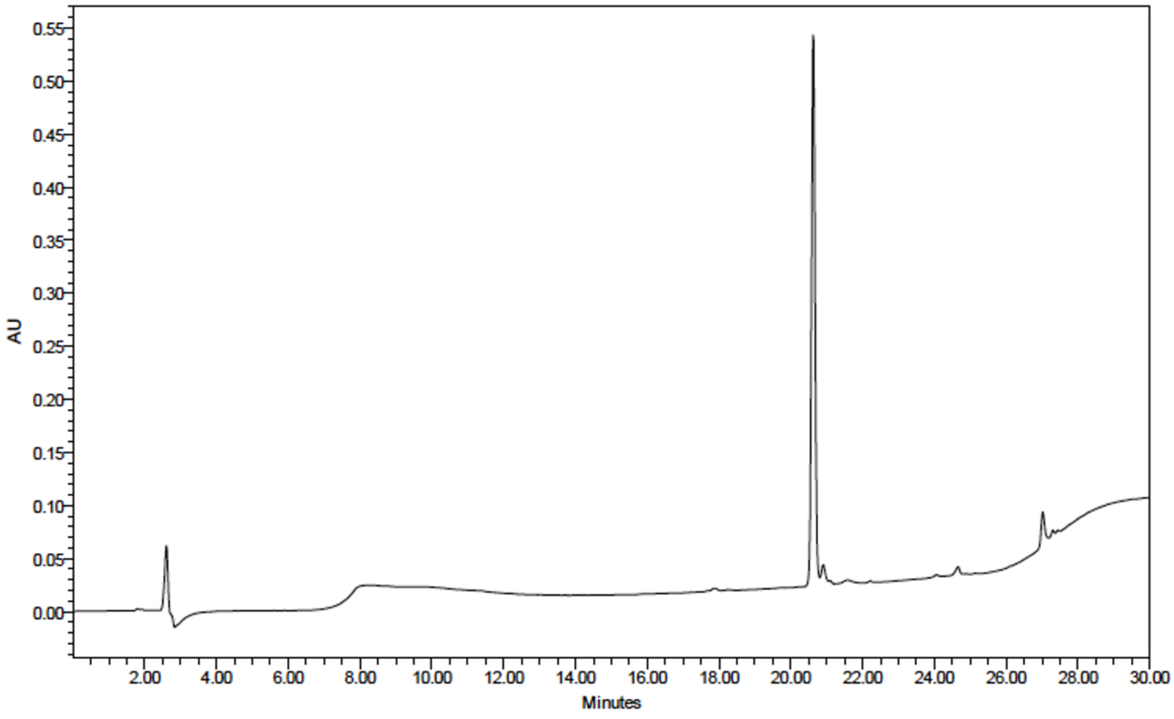

[C10]BP214 (**4**)

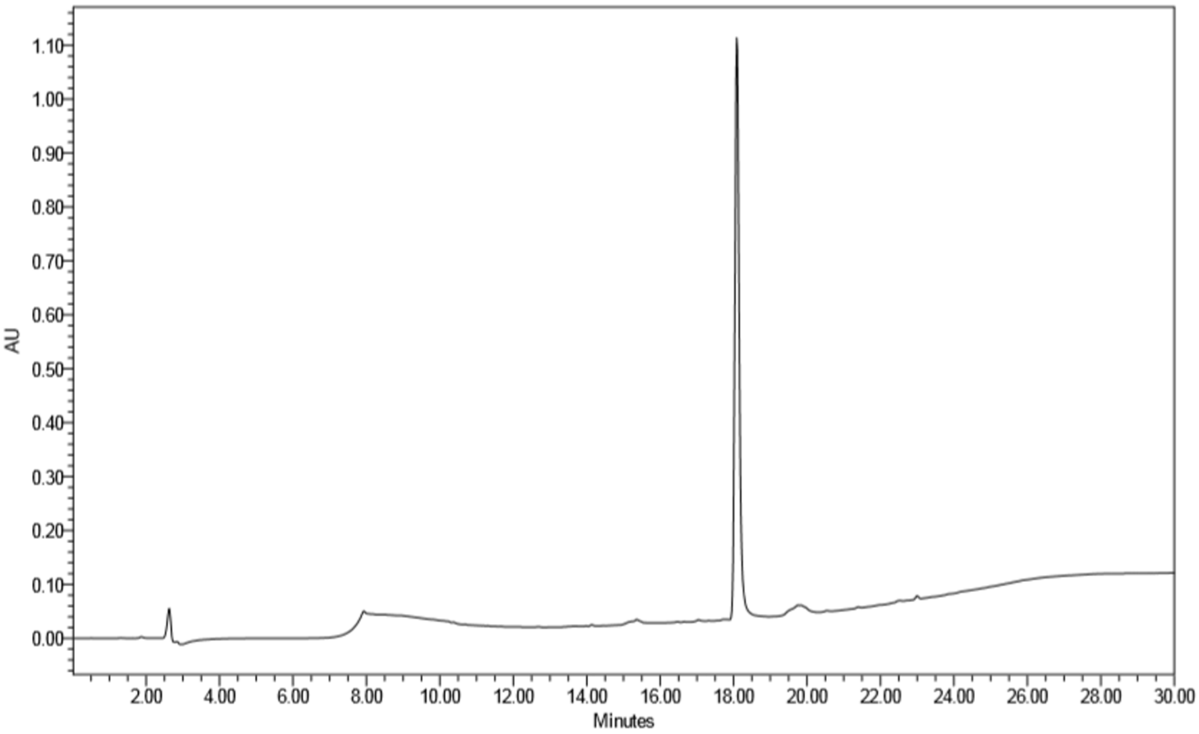

[C12]BP214 (**5**)

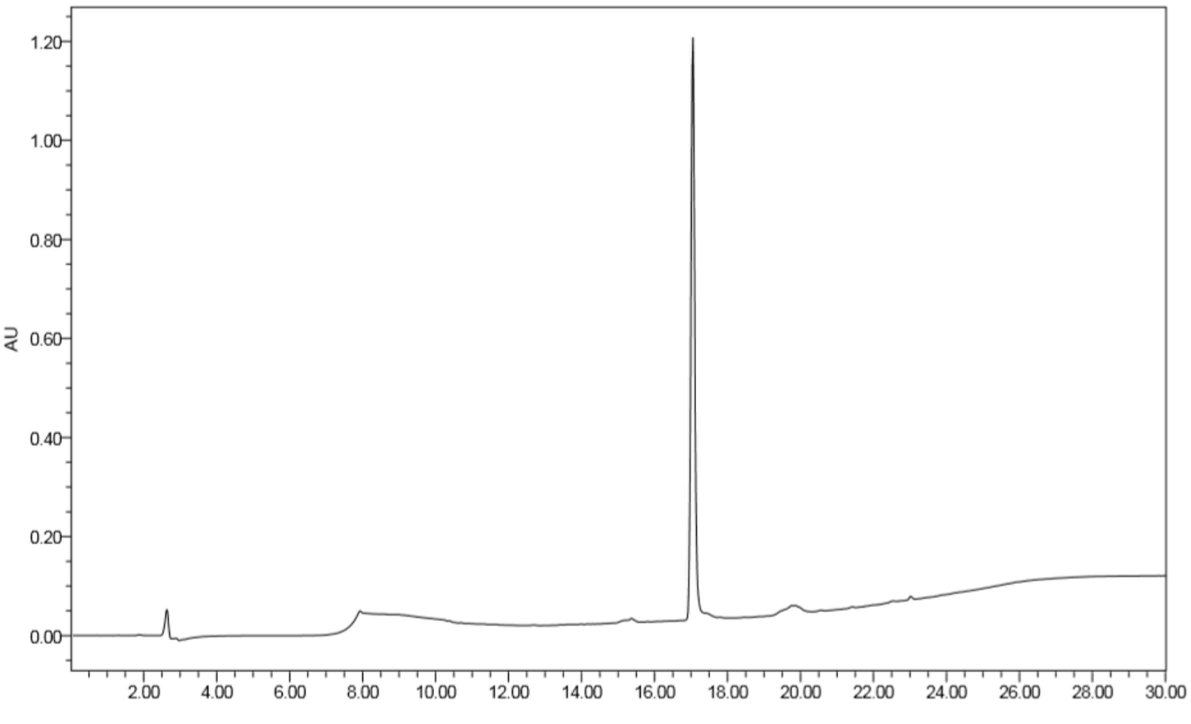

[C14]BP214 (**6**)

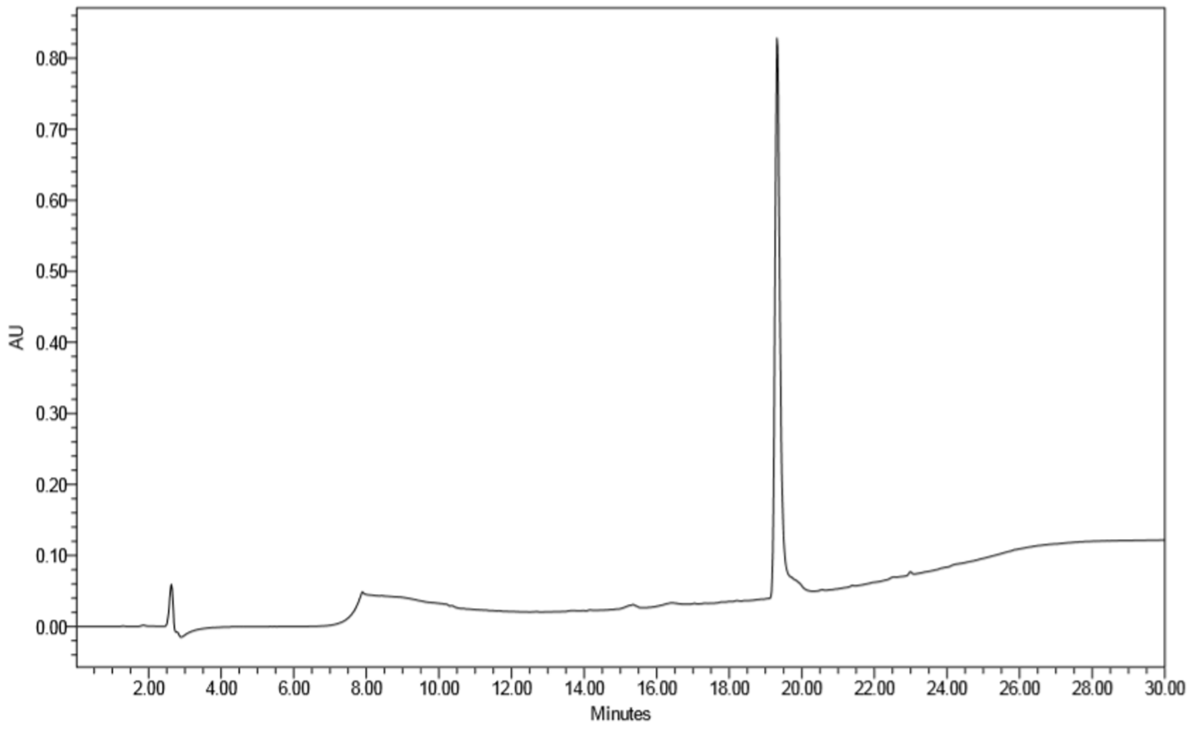

[*C3*]BP214 (7)

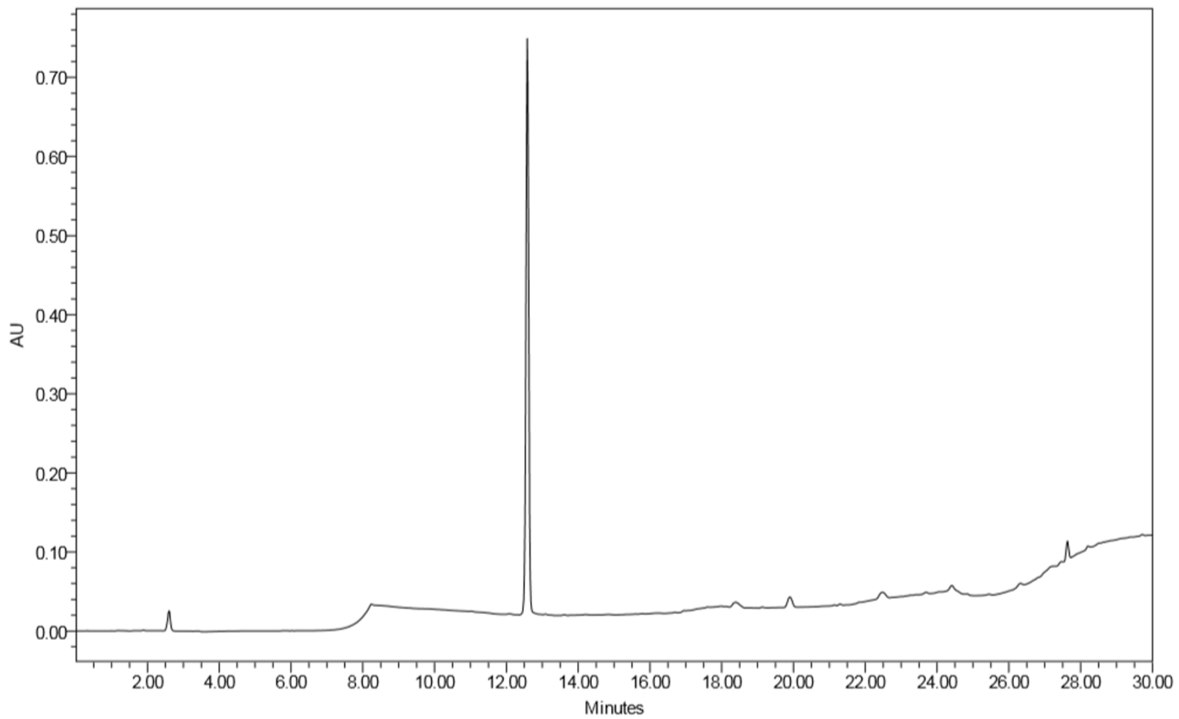

[*C4*]BP214 (8)

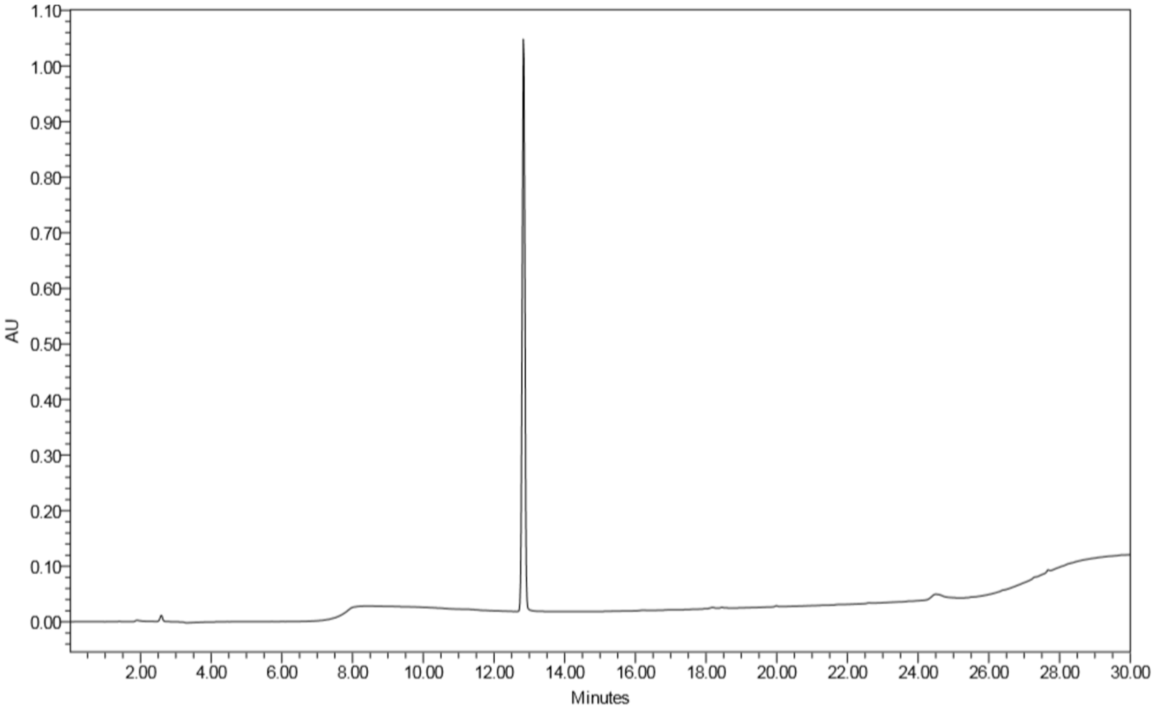

[*C5*]BP214 (9)

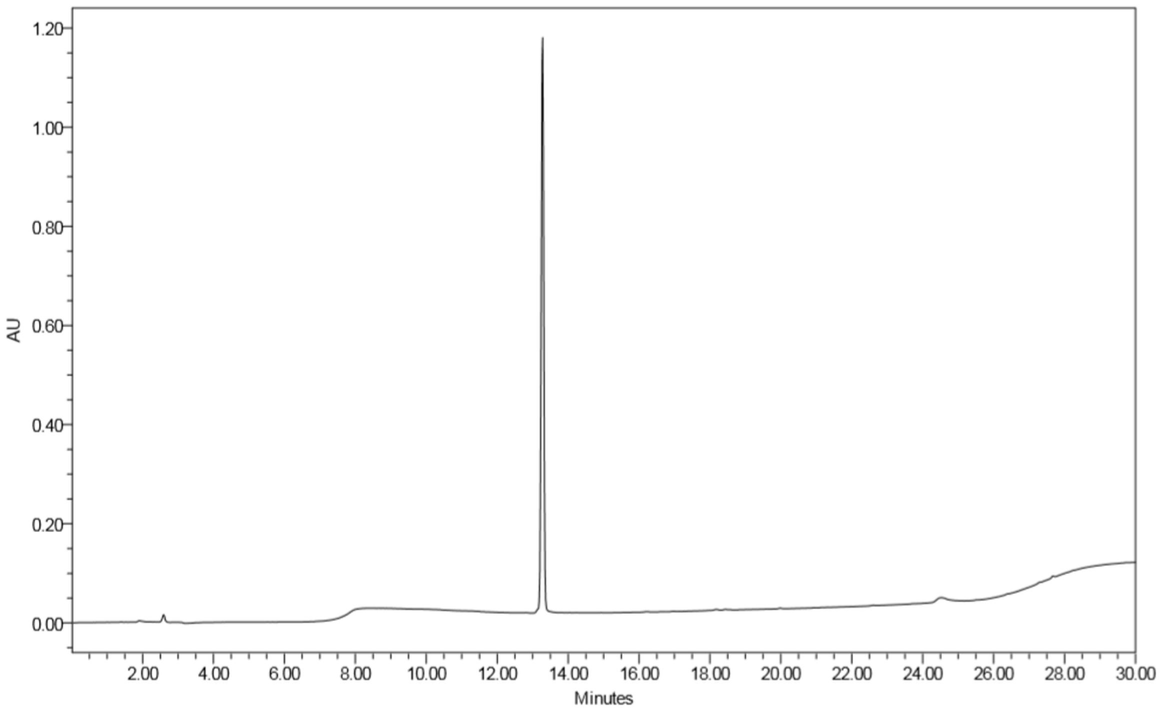

[(C_{10})*C*3]BP214 (10)

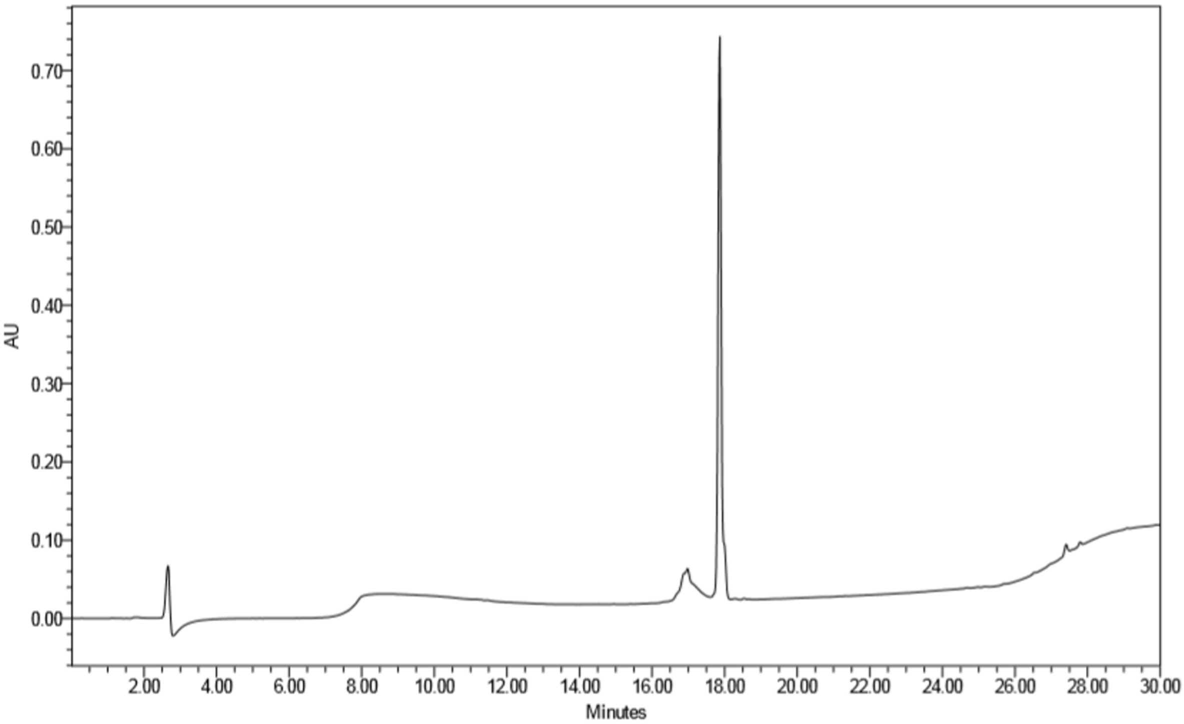

[(C_{12})*C*3]BP214 (11)

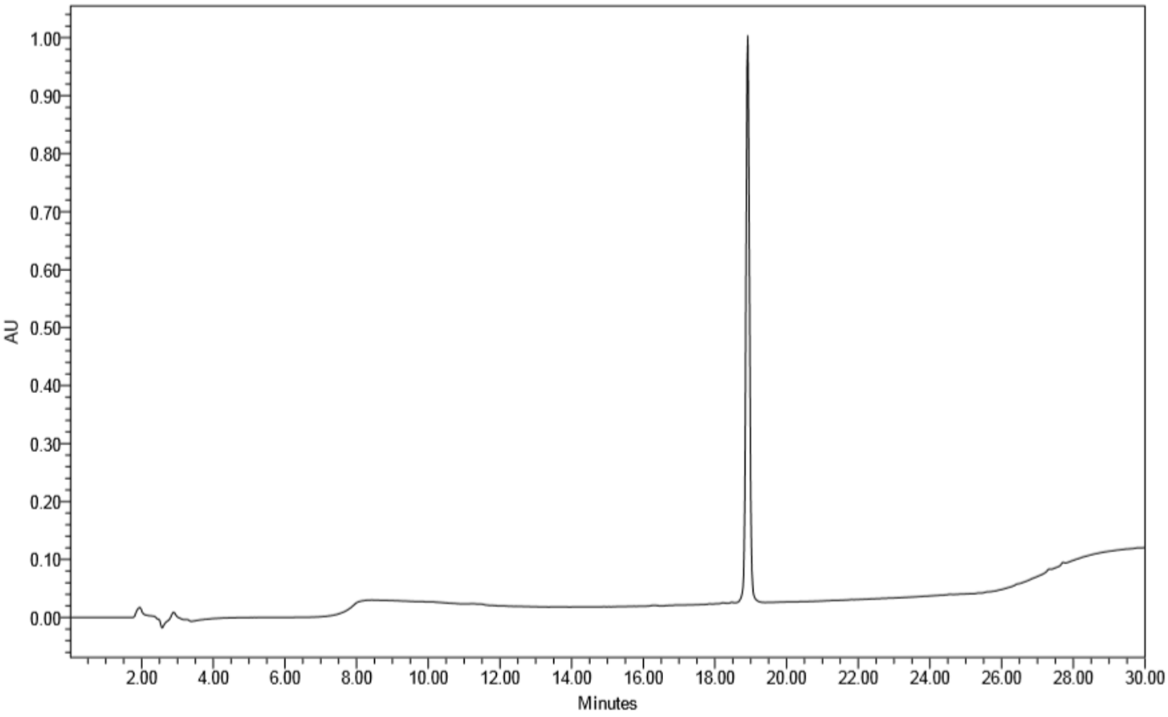

[(C_{14})*C*3]BP214 (12)

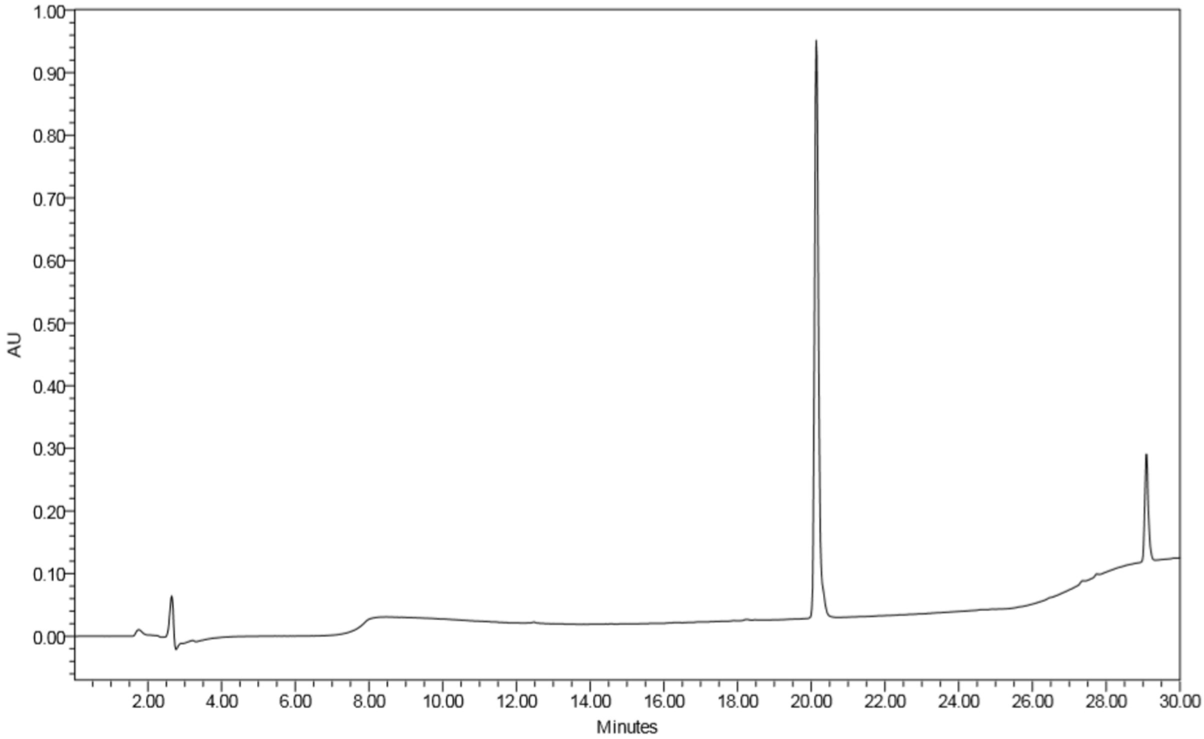

[(C_{10})*C*4]BP214 (13)

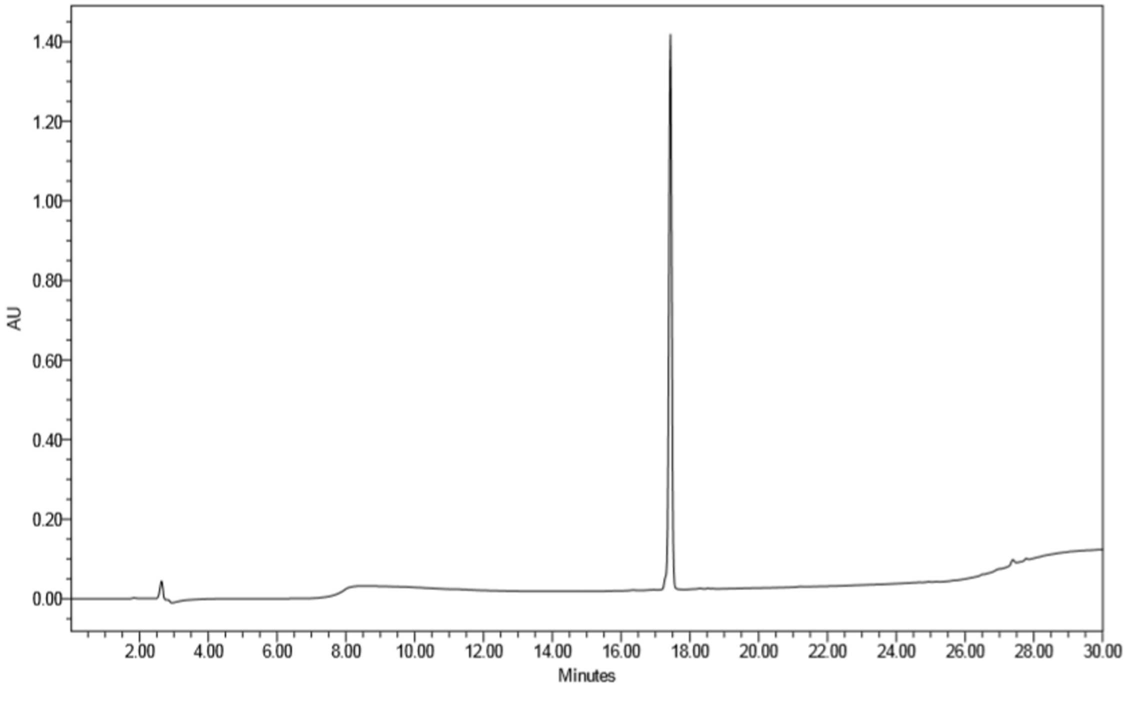

[(C_{12})*C*4]BP214 (14)

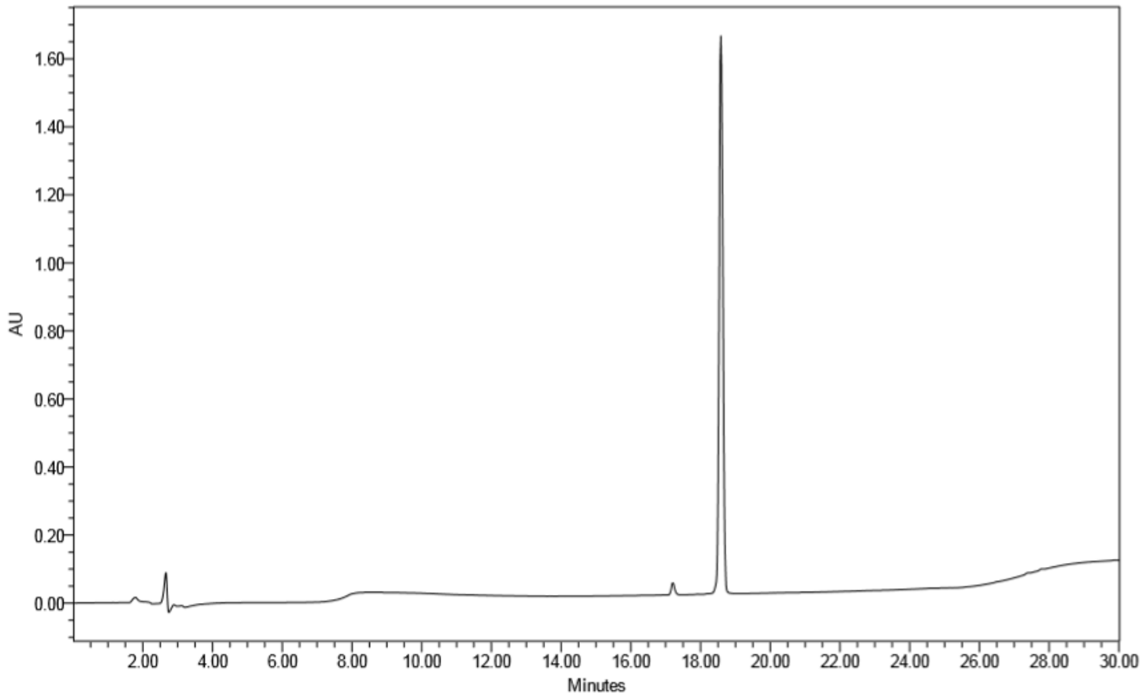

[(C_{14})*C*4]BP214 (15)

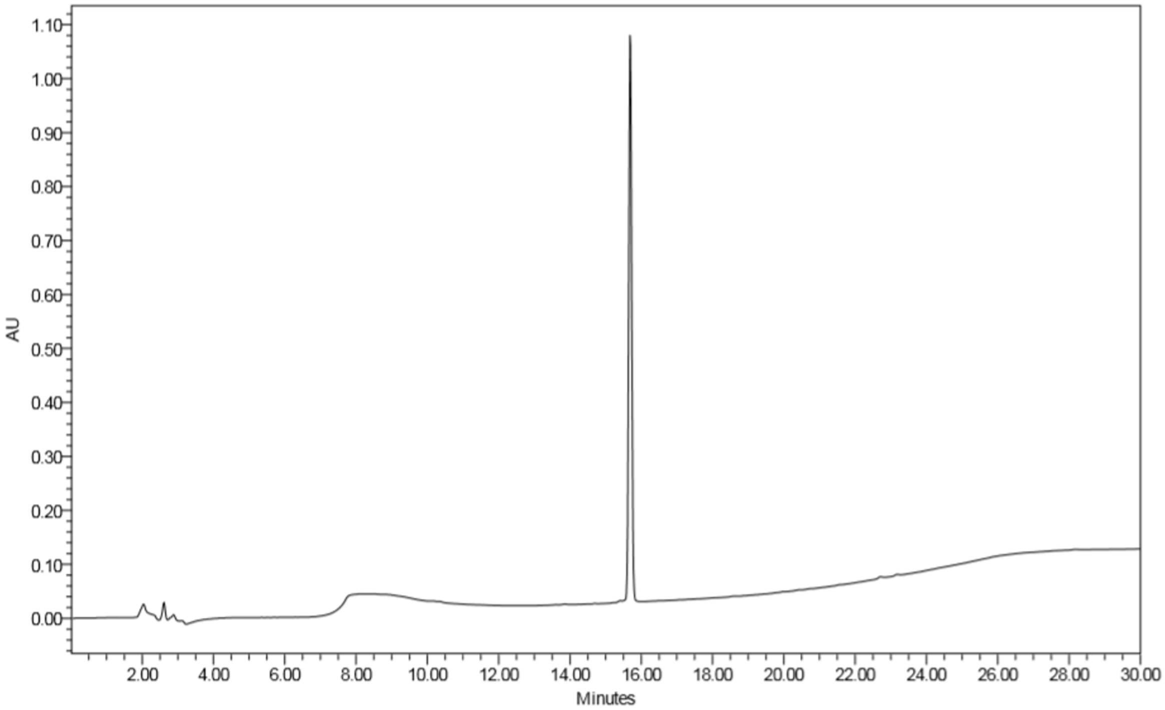

[(C_{10})*C*5]BP214 (16)

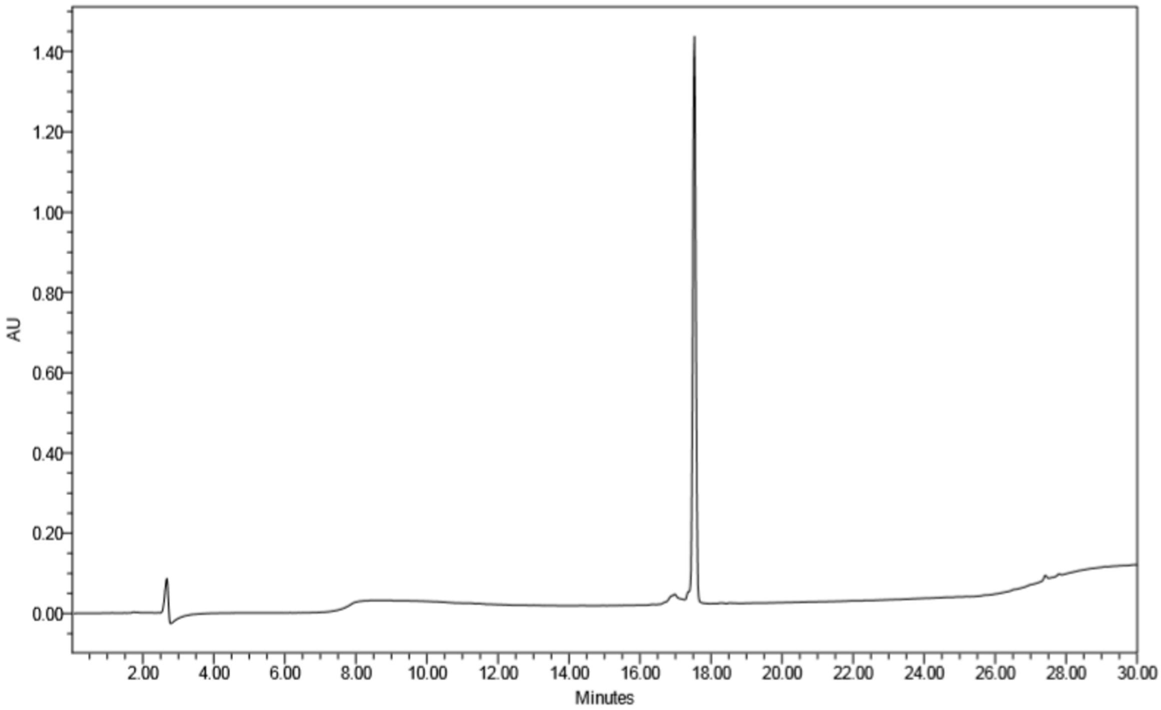

[(C_{12})*C*5]BP214 (17)

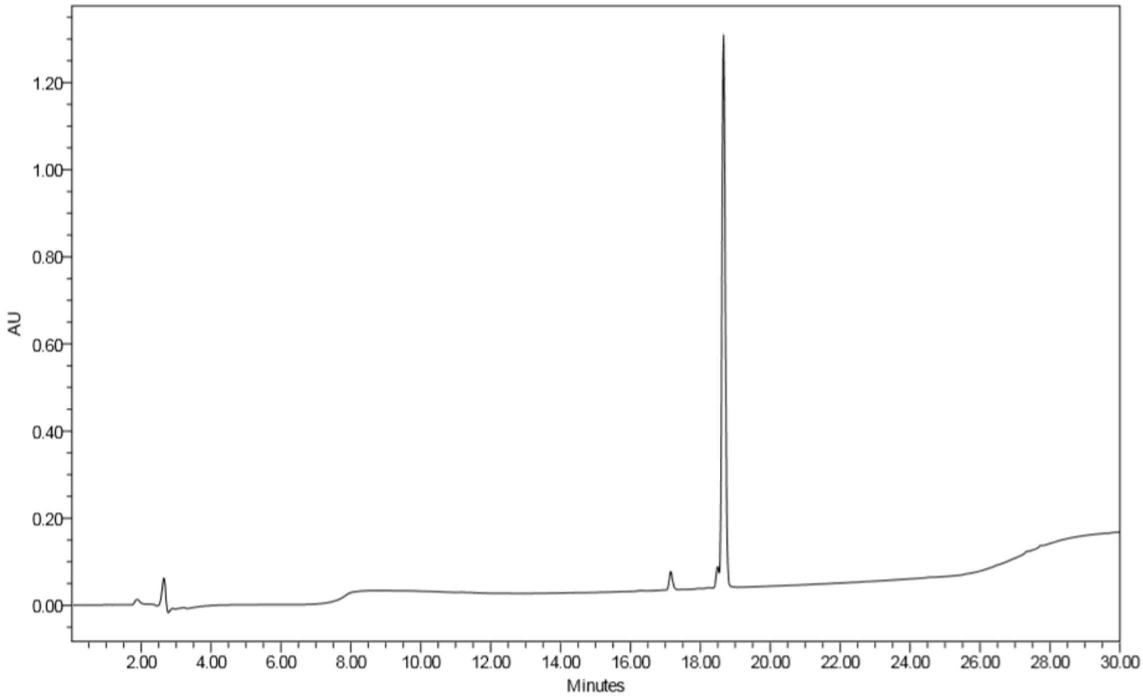

[(C_{14})*C*5]BP214 (18)

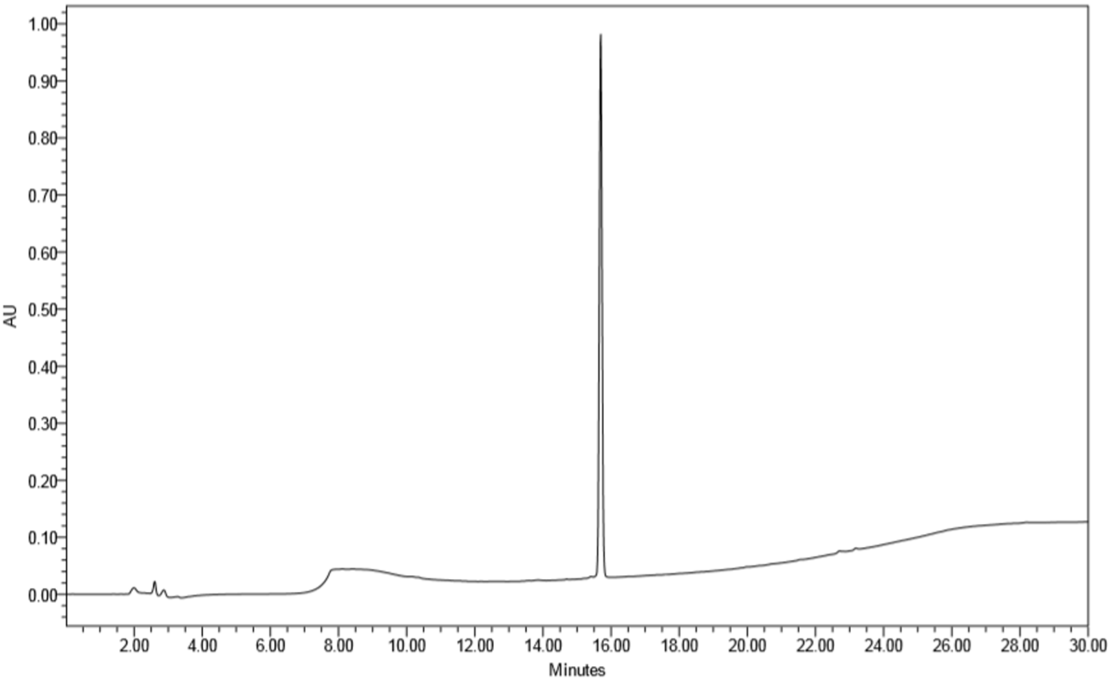

SIV: MALDI-TOF-MS spectra

BP214

Mw = 1448.91 Da

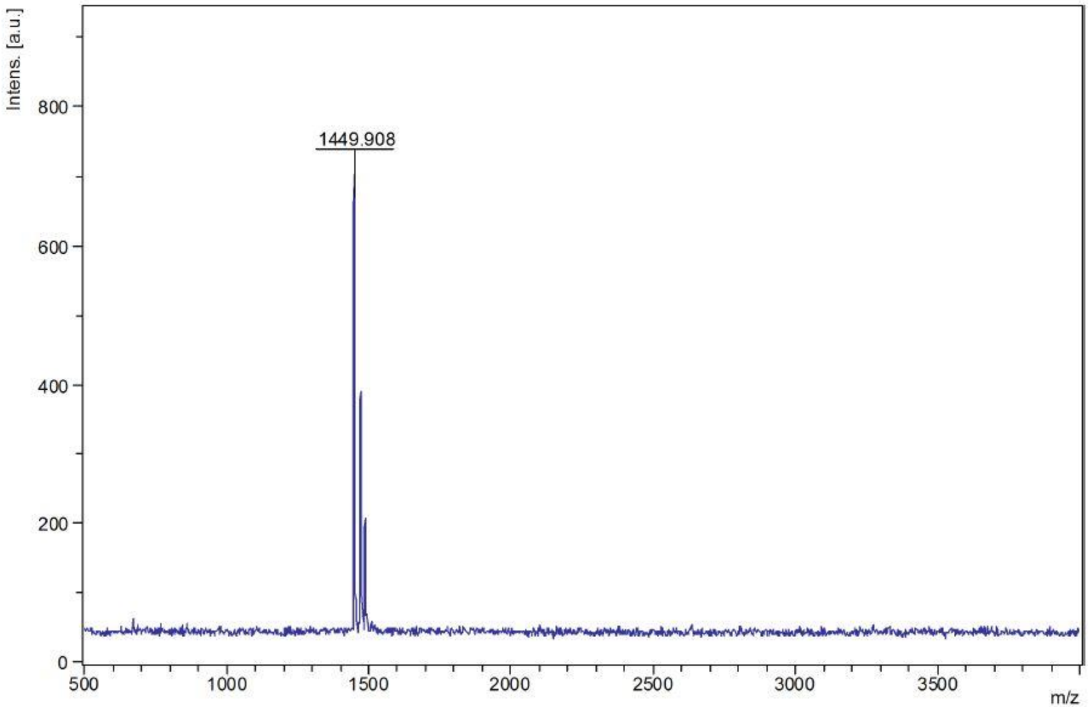

[C_4]BP214 (1)

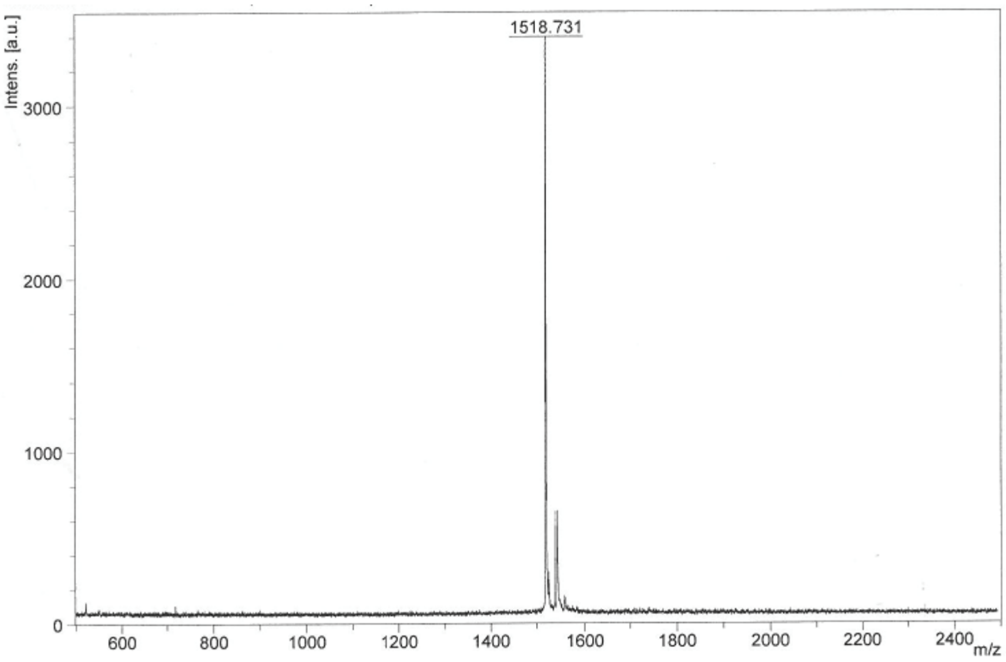

[C_6]BP214 (2)

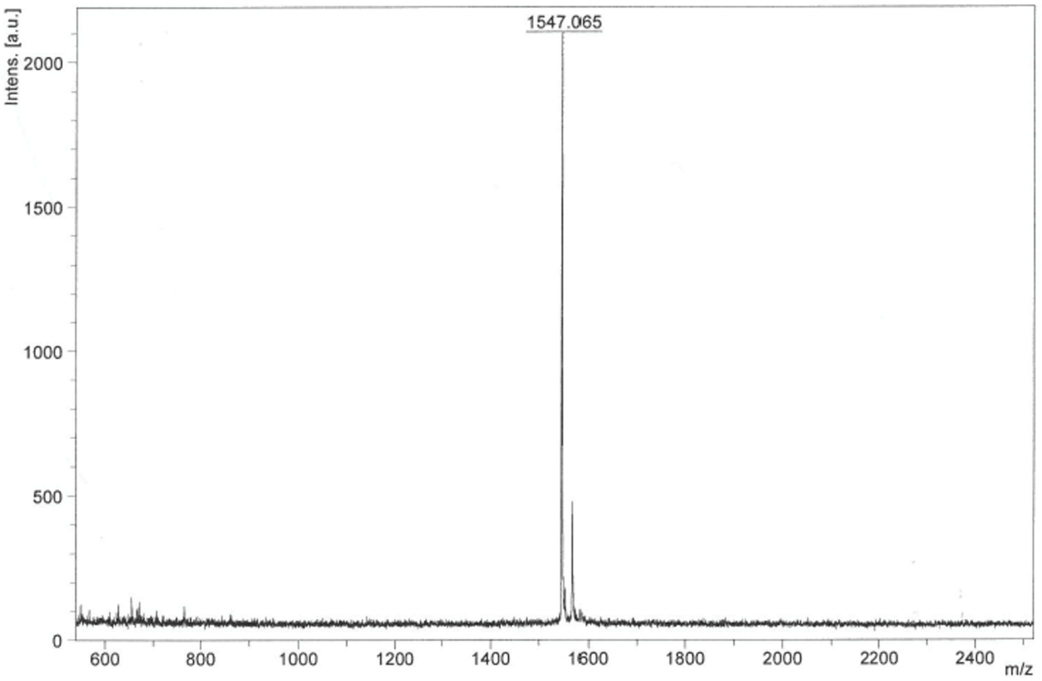

[C_8]BP214 (3)

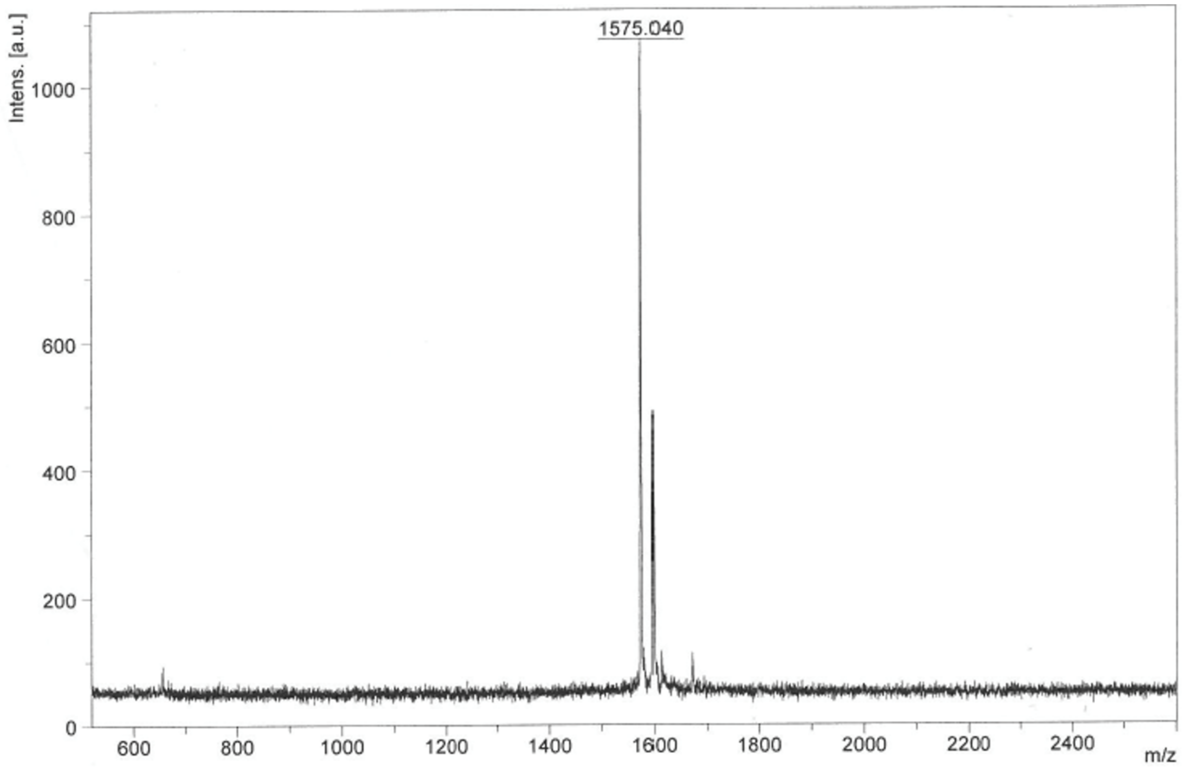

[C_{10}]BP214 (4)

Mw = 1603.17 Da

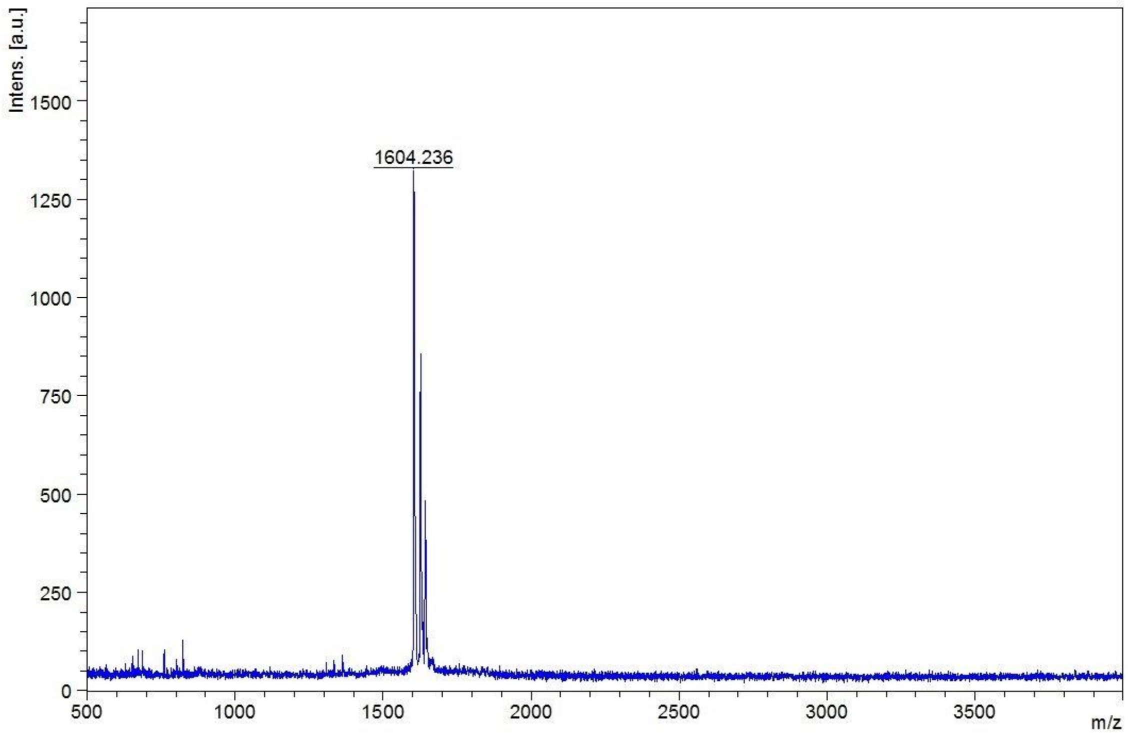

[C_{12}]BP214 (5)

Mw = 1631.22 Da

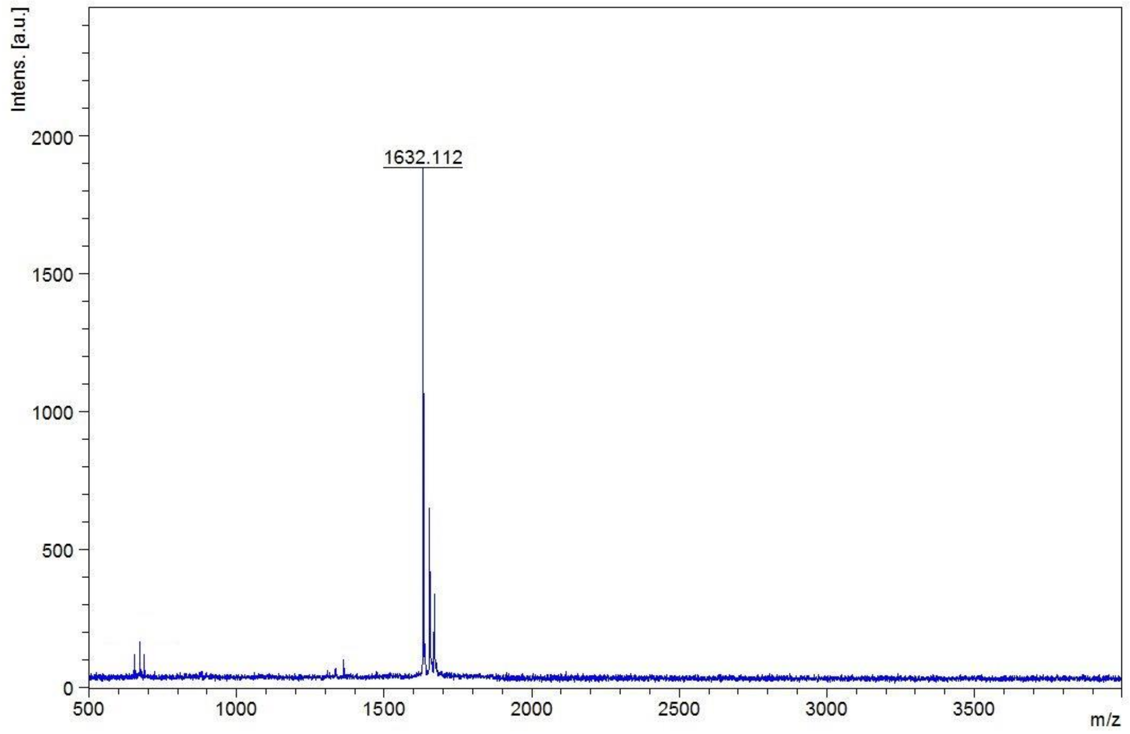

[C_{14}]BP214 (6)

Mw = 1659.27 Da

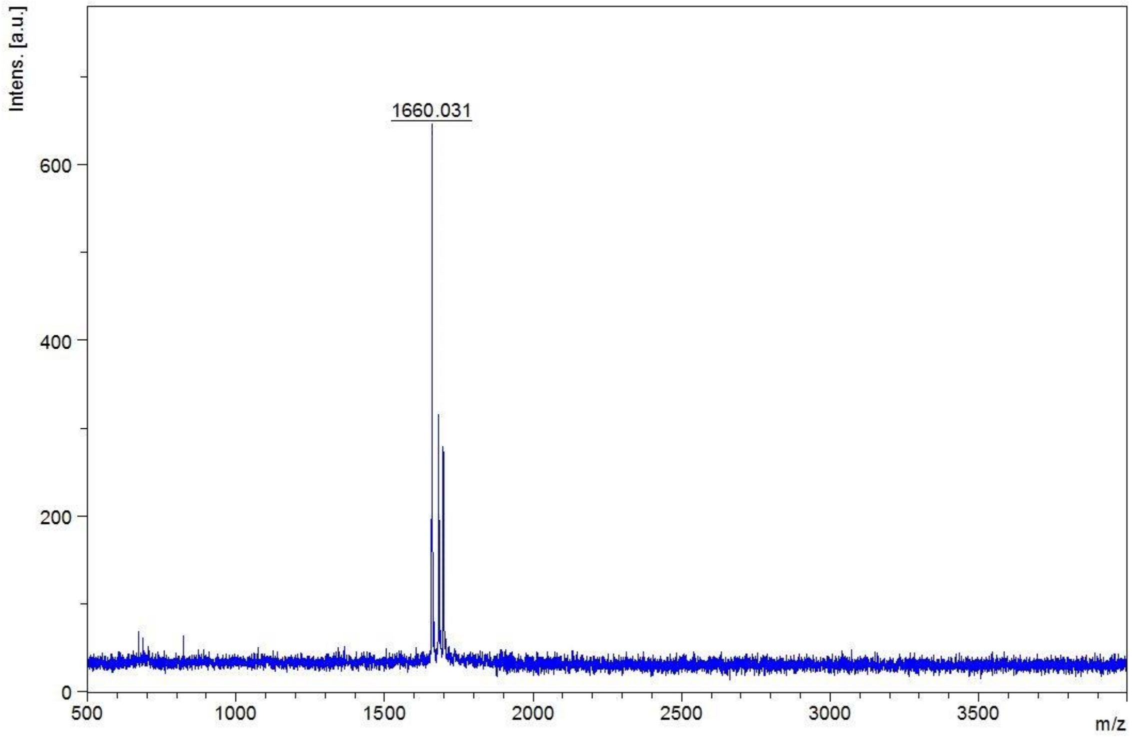

[*C*3]BP214 (7)

Mw = 1503.95 Da

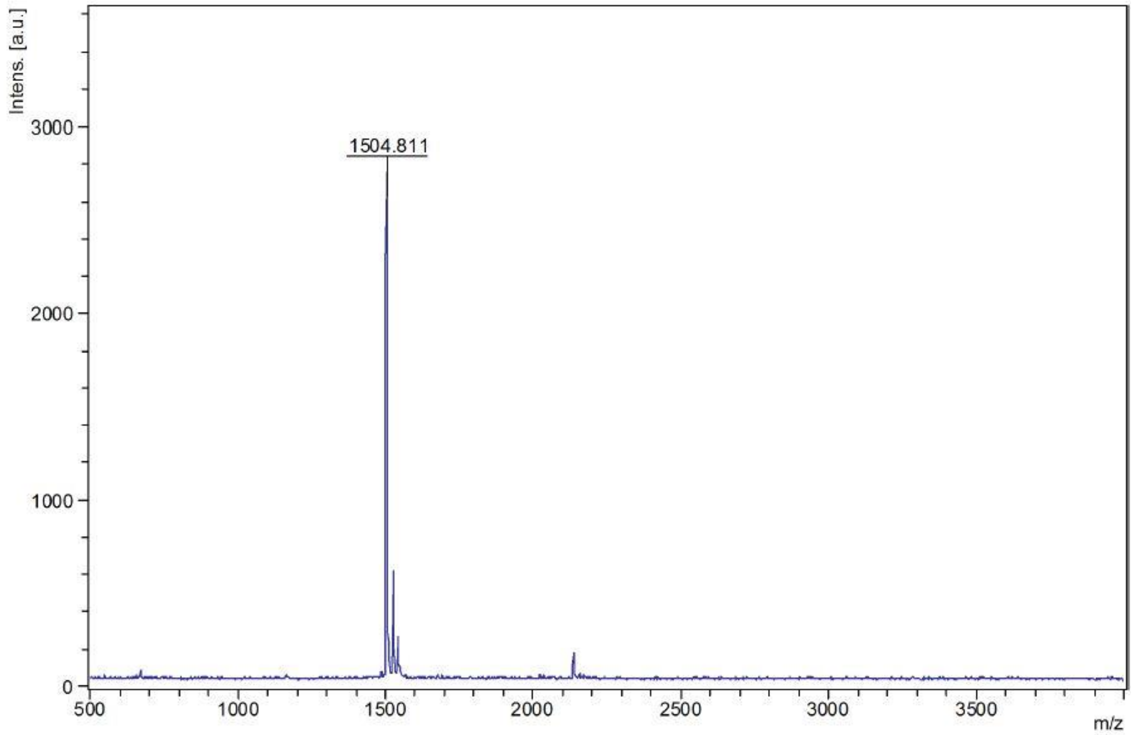

[*C*4]BP214 (8)

Mw = 1503.95 Da

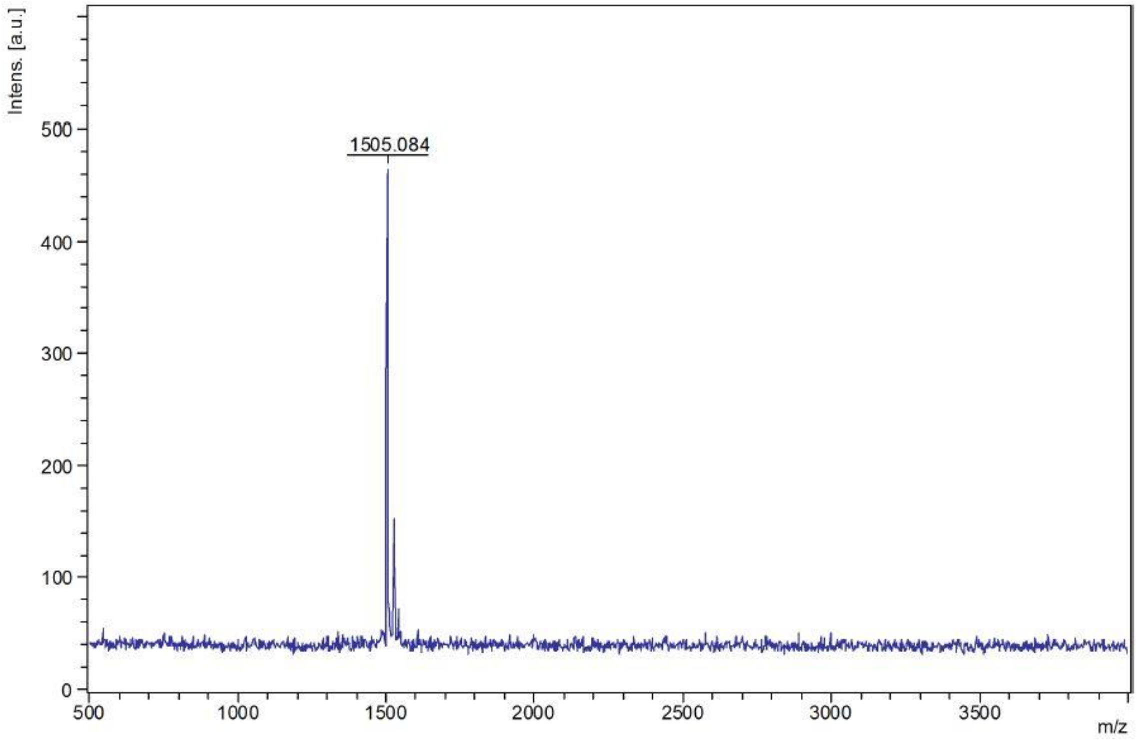

[*C*5]BP214 (9)

Mw = 1503.95 Da

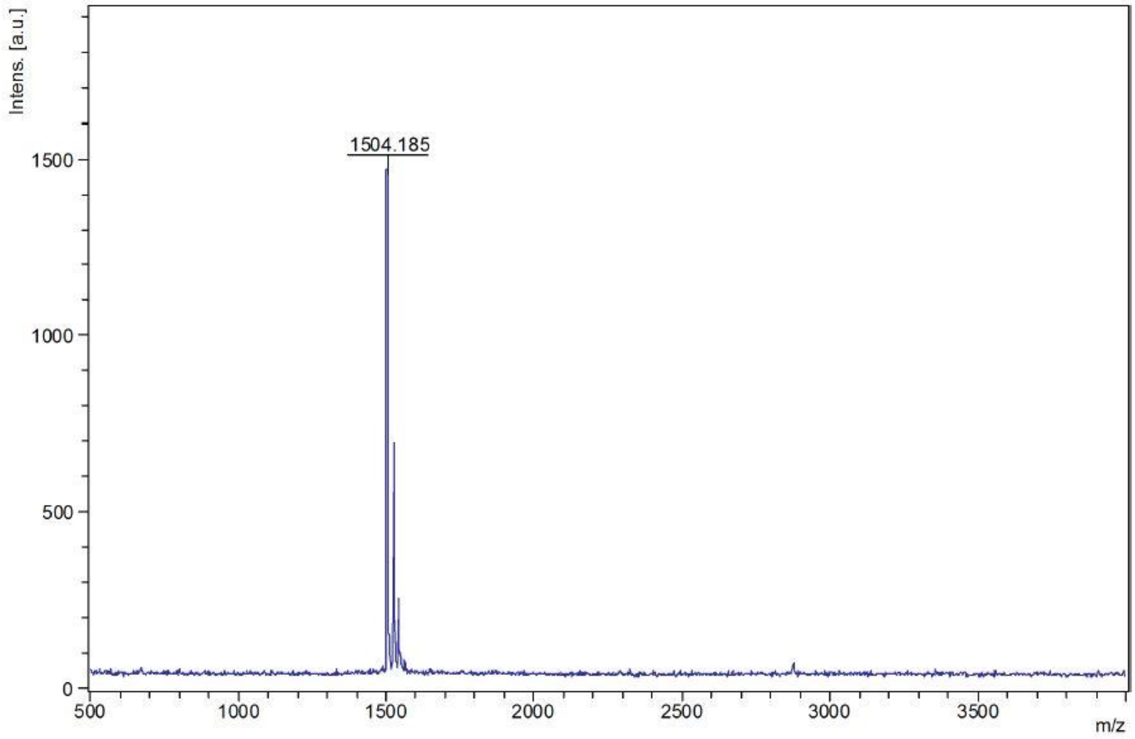

[(C_{10})*C*3]BP214 (10)

Mw = 1658.20 Da

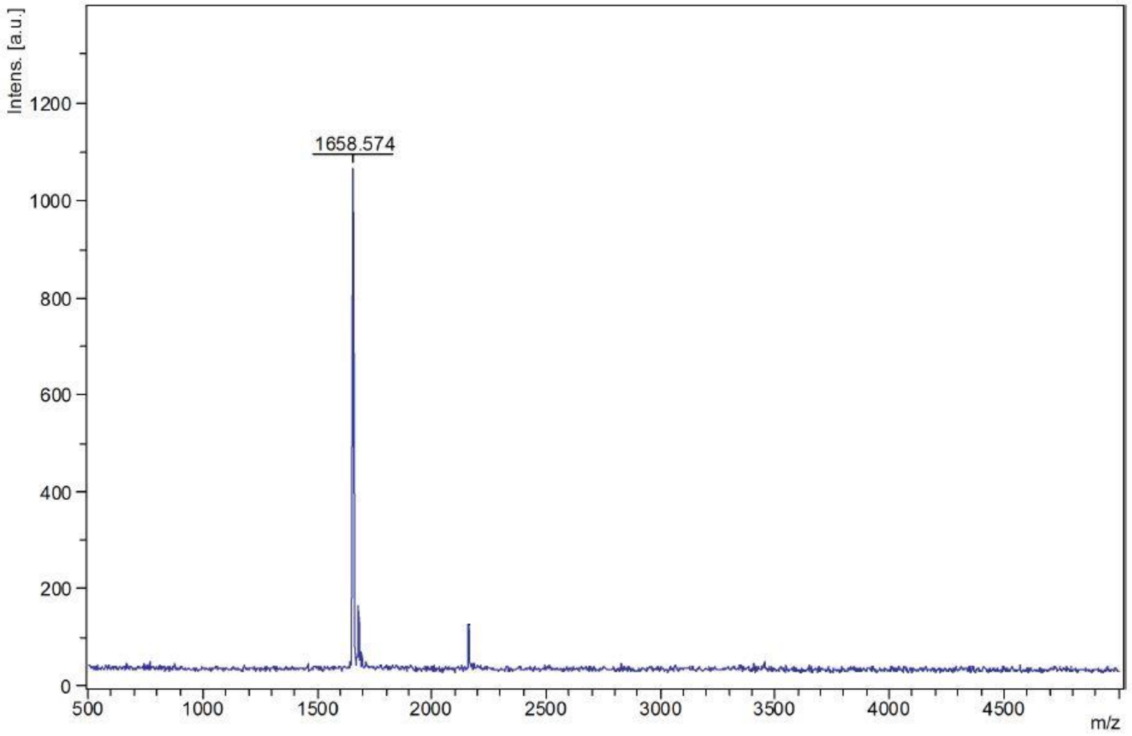

[(C_{12})*C*3]BP214 (11)

Mw = 1686.26 Da

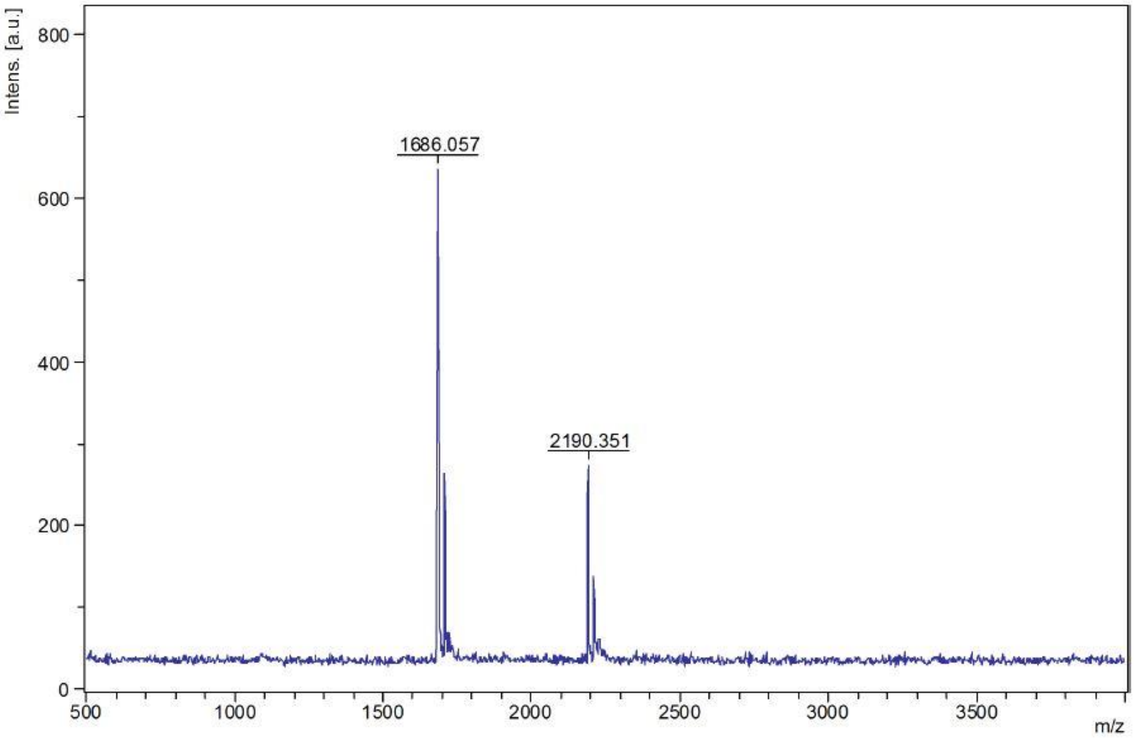

[(C_{14})*C*3]BP214 (12)

Mw = 1714.31 Da

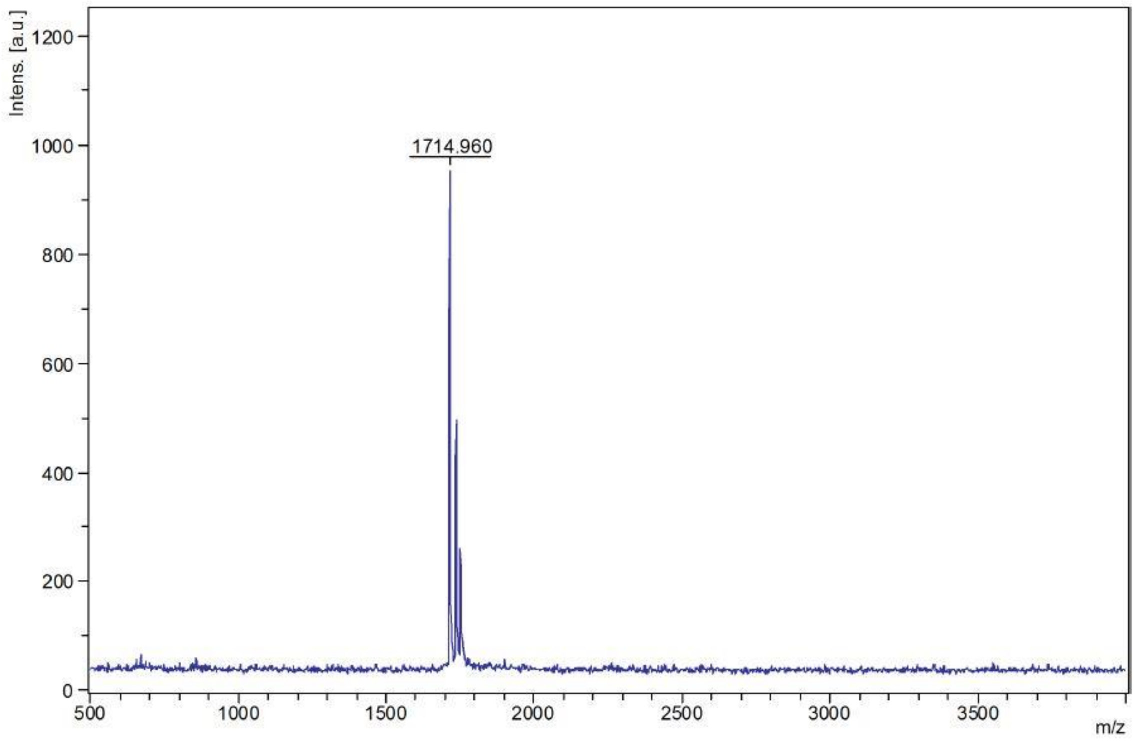

[(C_{10})*C*4]BP214 (13)

Mw = 1658.20 Da

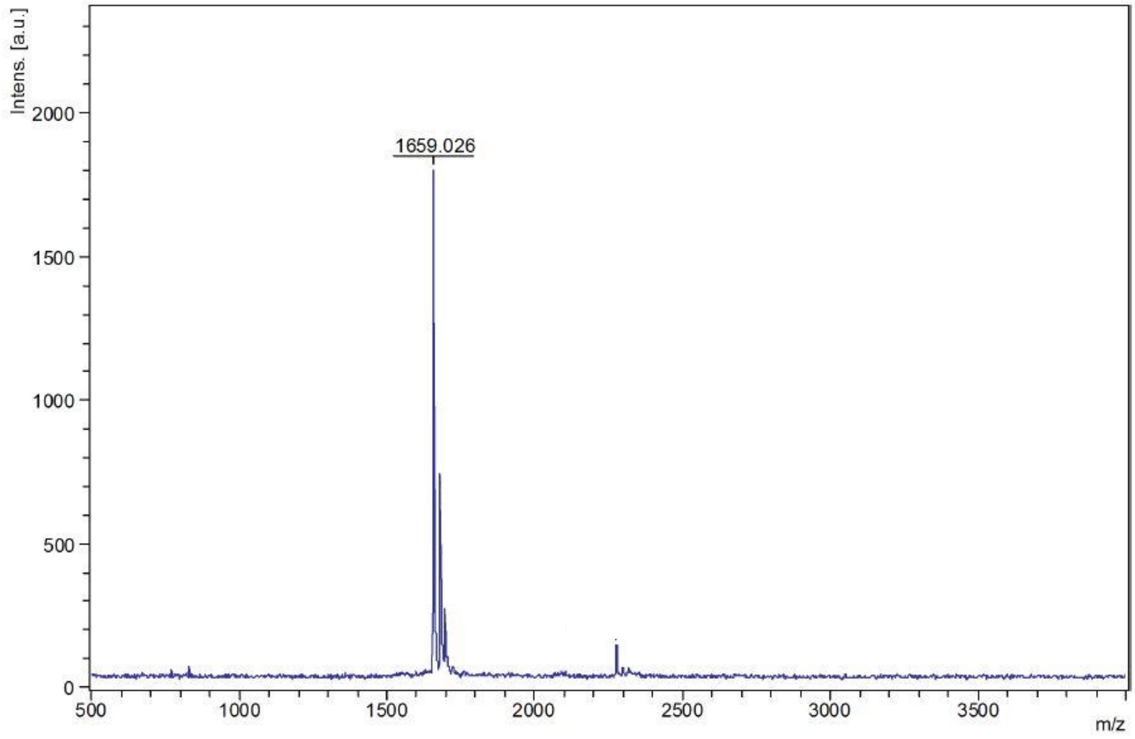

[(C_{12})*C*4]BP214 (14)

Mw = 1686.26 Da

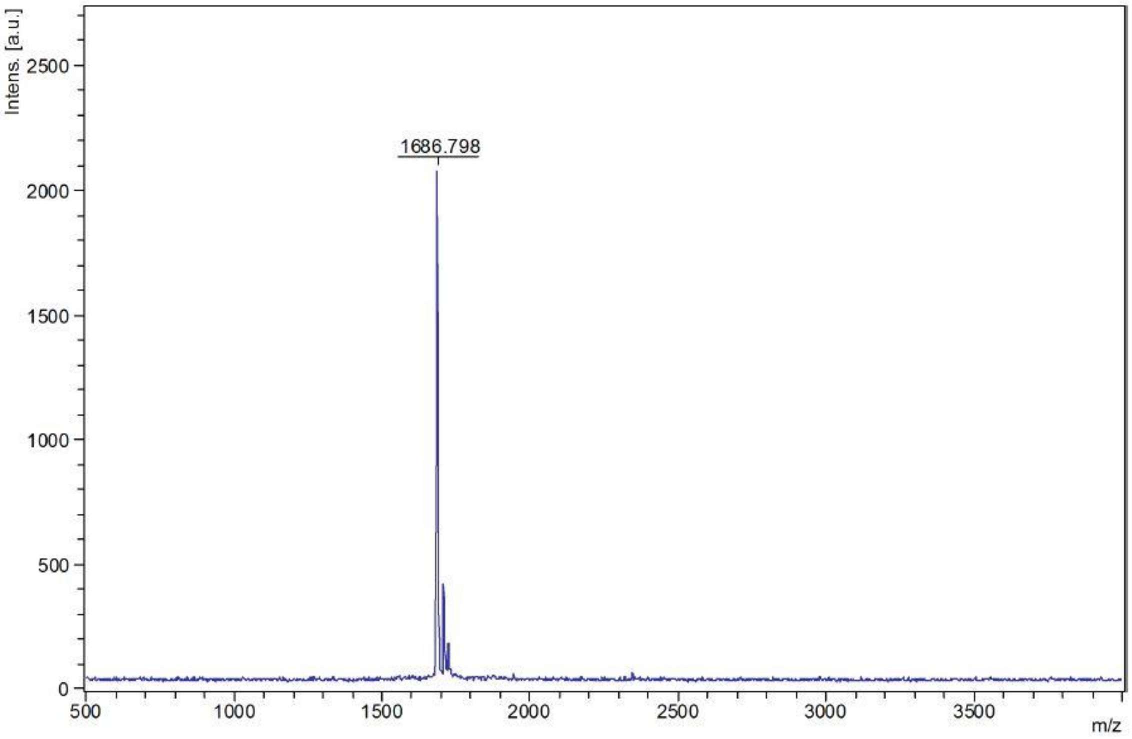

[(C_{14})*C*4]BP214 (15)

Mw = 1714.31 Da

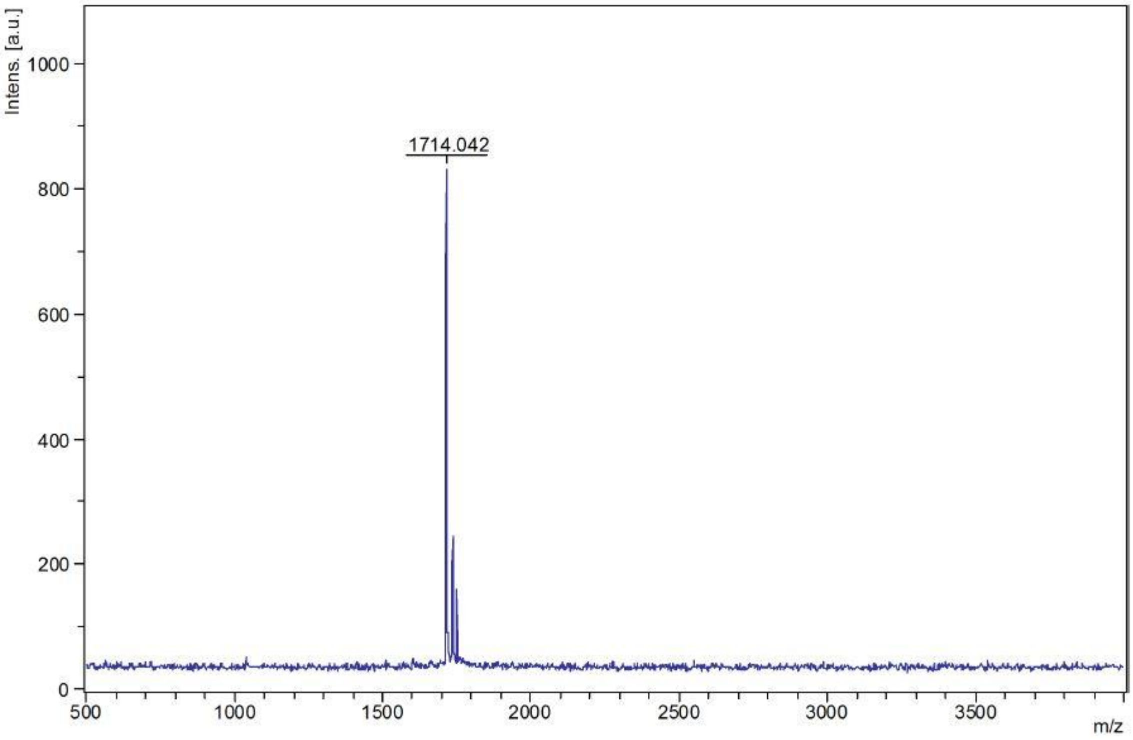

[(C_{10})*C*5]BP214 (16)

Mw = 1658.20 Da

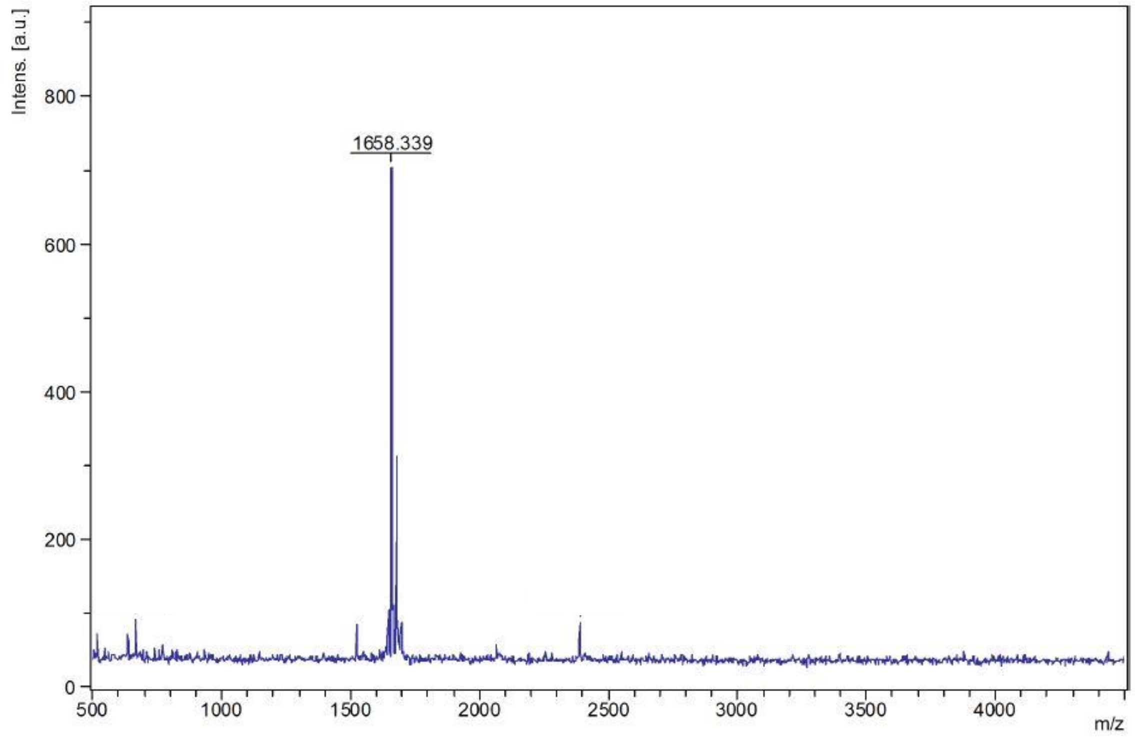

[(C_{12})*C*5]BP214 (17)

Mw = 1686.26 Da

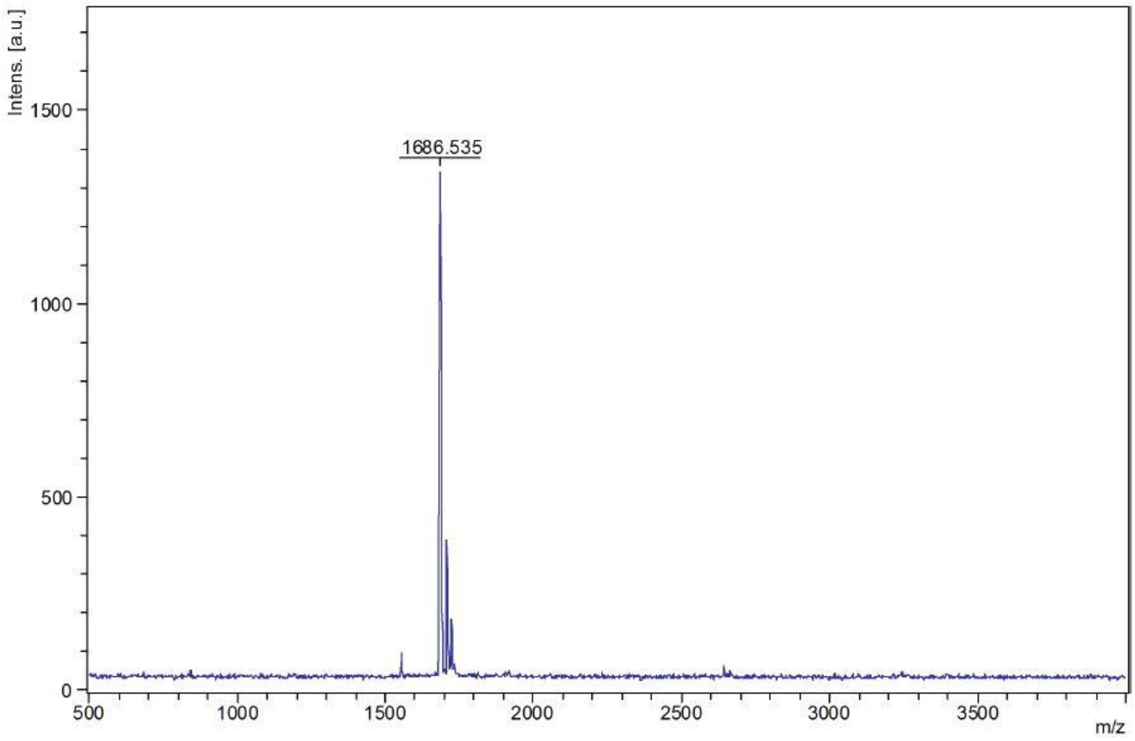

[(C_{14})*C*5]BP214 (18)

Mw = 1714.31 Da

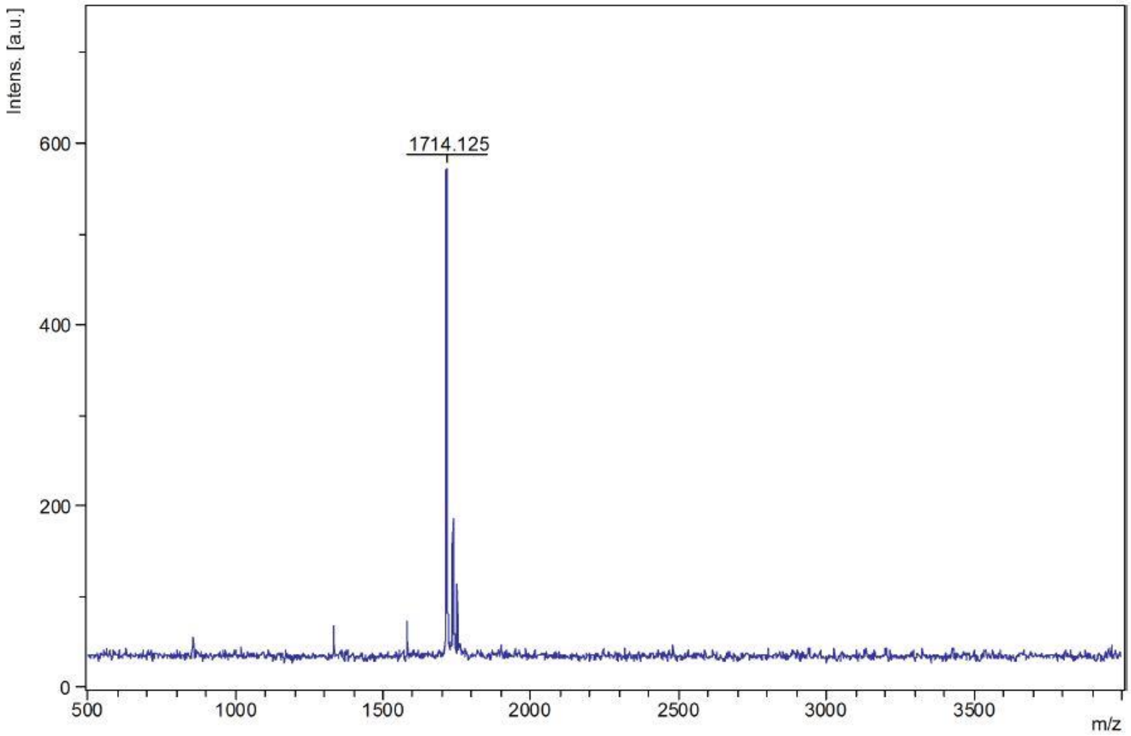

SV:

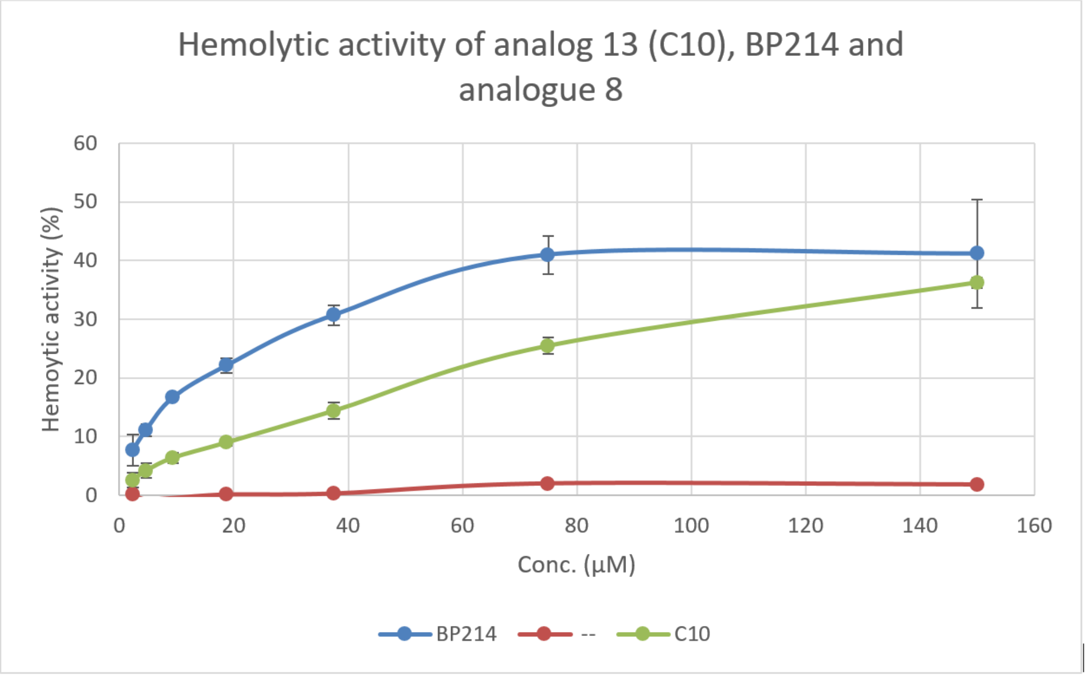

Hemolytic activity of analog 13 (C10), BP214 and analogue 8
Hemoytic activity (%)
60
50
40
30
20
10
0
0
20
40
60
80
100
120
140
160
Conc. (µM)
BP214
--
C10
